# The Influence of Mexican Hat Recurrent Connectivity on Noise Correlations and Stimulus Encoding

**DOI:** 10.3389/fncom.2017.00034

**Published:** 2017-05-10

**Authors:** Robert Meyer, Josef Ladenbauer, Klaus Obermayer

**Affiliations:** ^1^Department of Software Engineering and Theoretical Computer Science, Technische Universität BerlinBerlin, Germany; ^2^Bernstein Center for Computational NeuroscienceBerlin, Germany; ^3^Group for Neural Theory, Laboratoire de Neurosciences Cognitives, École Normale SupérieureParis, France

**Keywords:** noise correlations, neural coding, Mexican hat connectivity, spiking neural networks, pattern formation, primary visual cortex (V1), simulation models, stimulus encoding

## Abstract

Noise correlations are a common feature of neural responses and have been observed in many cortical areas across different species. These correlations can influence information processing by enhancing or diminishing the quality of the neural code, but the origin of these correlations is still a matter of controversy. In this computational study we explore the hypothesis that noise correlations are the result of local recurrent excitatory and inhibitory connections. We simulated two-dimensional networks of adaptive spiking neurons with local connection patterns following Gaussian kernels. Noise correlations decay with distance between neurons but are only observed if the range of excitatory connections is smaller than the range of inhibitory connections (“Mexican hat” connectivity) and if the connection strengths are sufficiently strong. These correlations arise from a moving blob-like structure of evoked activity, which is absent if inhibitory interactions have a smaller range (“inverse Mexican hat” connectivity). Spatially structured external inputs fixate these blobs to certain locations and thus effectively reduce noise correlations. We further investigated the influence of these network configurations on stimulus encoding. On the one hand, the observed correlations diminish information about a stimulus encoded by a network. On the other hand, correlated activity allows for more precise encoding of stimulus information if the decoder has only access to a limited amount of neurons.

## 1. Introduction

One of the fundamental problems in neuroscience is deciphering the neural code and understanding how the brain encodes sensory stimuli. During the past decades the analysis of neural coding has shifted from single cells to investigating population codes due to the development of multi-electrode recordings as well as improved theoretical models. An important aspect regarding population coding is whether neural responses are correlated, especially when driven by the same stimulus. In this case one speaks of *noise correlations* or *shared variability* (Cohen and Kohn, 2011; Hansen et al., 2012). Noise correlations have, for example, been widely observed in the visual cortex (Kohn and Smith, 2005; Martin and Schröder, 2013), where the magnitude of pairwise noise correlations decays with the distance between cell pairs (Smith and Kohn, 2008; Solomon et al., 2014). The underlying mechanisms and the role of noise correlations in information processing are, however, not well understood.

Noise correlations have often been explained by the large amount of shared inputs between neuron pairs (Gawne et al., 1996; Shadlen and Newsome, 1998) or, more recently, by cellular non-linearities (Doiron et al., 2016). Hansen et al. (2012) on the other hand hypothesized recurrent connectivity as one of the potential sources. This is a reasonable assumption considering that the vast majority of connections does not come from the sensory periphery or higher cortical areas, but originates within the visual cortex and projects locally (Markov et al., 2011).

Although work by Renart et al. (2010) showed that a balance between excitation and inhibition among recurrent connectivity can lead to the opposite effect and cause decorrelation of activity, more recent studies indicate that this phenomenon depends on the spatial structure of recurrent connections (Rosenbaum and Doiron, 2014). Rosenbaum and Doiron (2014) demonstrated analytically using a continuous rate model that homogeneous, balanced activity cannot be maintained in networks with Gaussian Mexican hat connectivity profiles, where the spatial reach of recurrent excitatory connections is on average shorter than that of recurrent inhibitory connections. Moreover, Hansel and Sompolinsky (1998) have shown that such connections can lead to the emergence of spatial patterns of activity in form of bumps (also see Roxin et al., 2006, for a more recent summary of the findings). This type of activity is termed *marginal* phase. They further demonstrated that under certain conditions the bumps move across the spatial extent of the network.

In this study we investigate the hypothesis that recurrent connections in the form of a Mexican hat configuration can lead to noise correlations by destabilizing homogeneous, balanced activity, and enabling temporally dynamic, spatially inhomogeneous patterns of activity. Motivated by aforementioned theoretical results we first study the dynamics of two-dimensional networks of spiking model neurons for different lateral interaction kernels and spatially unstructured homogeneous input. We find that a Mexican hat configuration leads to the emergence of dynamic stripe or bump patterns that in turn yield noise correlations. The dynamic spatial heterogeneities produce joint modulations of firing rates on the individual neuron level. Accordingly, we observed oscillatory spatial modulations of positive and negative noise correlations.

We then study the interaction of the neural dynamics with structured input, which is derived from an orientation map model of primary visual cortex. We find interactions among the input dynamics and the marginal phase. In particular, the oscillatory pattern of noise correlations with distance vanishes and we observe a linear decay instead. In addition, we find a suppression of noise correlations with input strengths. Finally we ask, what effects the observed noise correlations have on the quality of the neural code given structured inputs. We investigate whether Mexican hat connectivity with noise correlations would be a better or worse choice than inverse Mexican hat connectivity with decorrelated firing patterns. We find that correlations are detrimental to encoding quality in terms of Fisher information. However, in case readout neurons are sub-sampled, Mexican hat networks show a better encoding performance despite the noise correlations.

## 2. Materials and methods

### 2.1. Model description

Our model is based on a primary visual cortex network developed by Mariño et al. (2005) and Stimberg et al. (2009). Here we provide a brief overview only. For details including the equations describing the underlying dynamics please refer to the Supplementary Material.

The model consisted of *N* = *N*_*E*_ + *N*_*I*_ excitatory and inhibitory neurons of the adaptive exponential integrate and fire (AEIF) type (Brette and Gerstner, 2005). We assumed a ratio between inhibitory to excitatory neurons of 1–4 (Beaulieu et al., 1992). The AEIF model is frequently applied in computational neuroscience due to its efficiency and good fits to experimental data (Brette and Gerstner, 2005; Jolivet et al., 2008). It can exhibit a very rich set of dynamics including spike frequency adaptation (SFA) (Naud et al., 2008; Touboul and Brette, 2008) which is an essential ingredient for non-stationarity of bump attractors (cf. Hansel and Sompolinsky, 1998; Roxin et al., 2006; and Supplementary Material). We chose moderate sub-threshold and spike-triggered adaptation for excitatory neurons (*a*_*E*_ = 2.0 nS, *b*_*E*_ = 50 pA). To account for the fact that adaptation is much weaker in inhibitory neurons (Nowak et al., 2003), for inhibitory cells adaptation parameters were set to one tenth of the excitatory values (*a*_*I*_ = 0.2 *nS*, *b*_*I*_ = 5 *pA*).

#### 2.1.1. Topology and coupling

**Figure 1:**
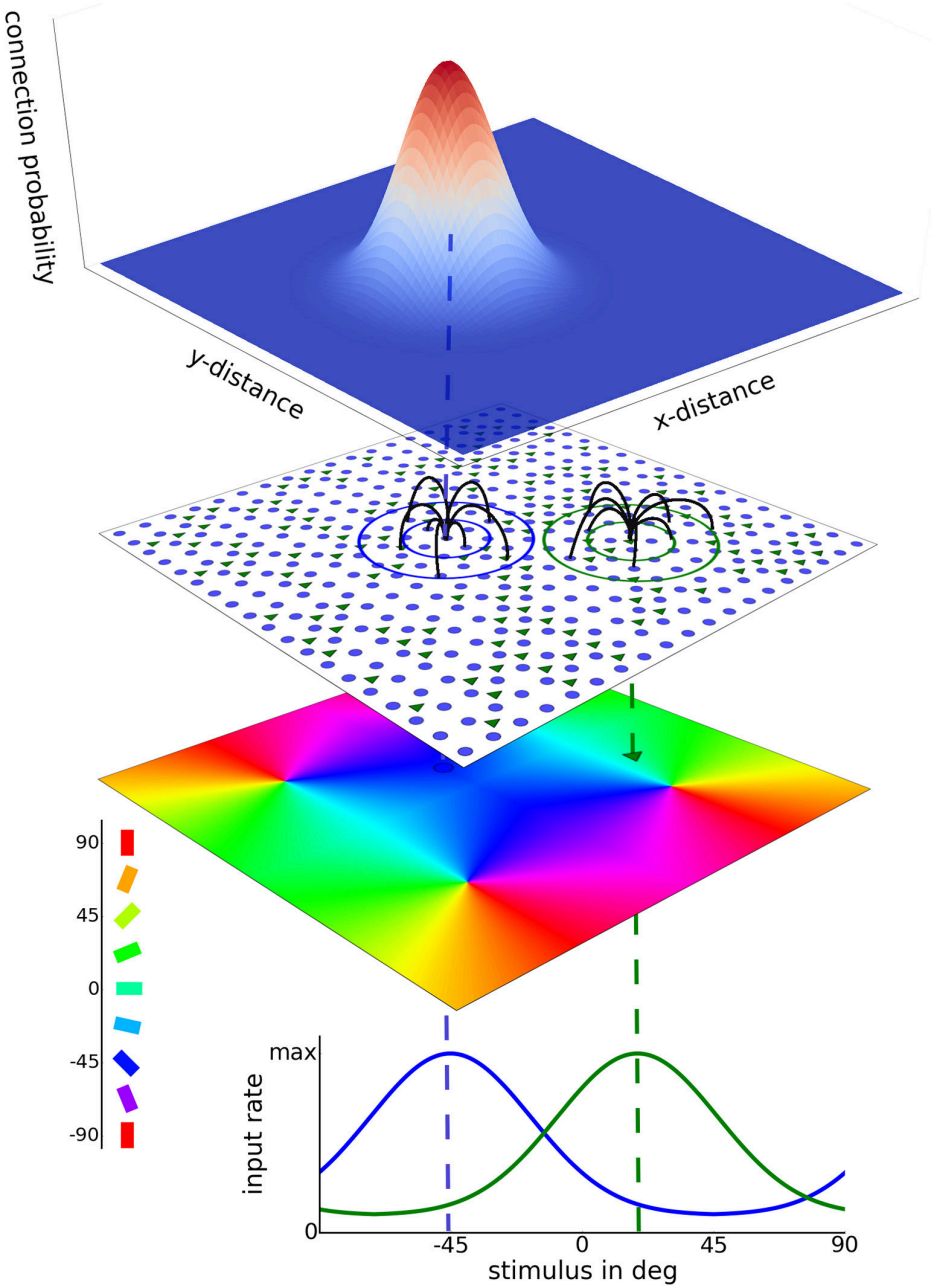
**2D model network architecture**. A layer of excitatory neurons (blue dots) and inhibitory neurons (green triangles) receives afferent as well as recurrent lateral input. Excitatory neurons are placed on a regular grid and inhibitory neurons are assigned to randomly chosen grid positions. Examples of lateral connections are depicted by the black lines. These connections are sampled from Gaussian probability kernels depending on cell distance, as depicted at the top. In the case of heterogeneous input a preferred orientation is assigned to each model neuron according to its position in the artificial orientation map with 4 pinwheels (bottom colored sheet). Afferent stimulation is realized as independent Poisson spike trains. A circular Gaussian tuning curve with a width of 27.5° determines the afferent rate for each neuron as a function of the presented stimulus orientation. Input tuning curves for two different cells are shown at the bottom. In case of homogeneous input, each neuron is driven by afferent Poisson stimulation with the same rate.

We investigated two-dimensional network models where neurons were placed on a grid. Additional results from one-dimensional networks are presented in the Supplementary Material. Excitatory neurons were regularly and evenly spaced on the grid, while the positions of the inhibitory neurons were chosen randomly from a uniform distribution defined over the network space. Distances in our network are given in pixels (px), with 1 px corresponding to 15 µm. The conversion factor is calculated from the average pinwheel distance in cat primary visual cortex (Stimberg et al., 2009). Thus, a 100 px × 100 px network with 4 orientation pinwheels corresponds to a 1.5 mm × 1.5 mm piece of primary visual cortex. Such a two-dimensional network topology is depicted in Figure 1.

Each neuron received *K*_*E*_ = 400 excitatory and *K*_*I*_ = 200 inhibitory recurrent connections. These synaptic connections were drawn from Gaussian probability distributions defined over the Euclidean distances *d*_*ij*_ between two model neurons *i* and *j* (periodic boundary conditions):

(1) $$\begin{array}{l} p\left(i,j\right)=\left\{ \begin{array}{ll} 0\; & \text{for }i=j(\text{no}\\ \; & \text{self}-\text{connections})\\ 1/{\left(\sqrt{2\pi }\sigma_{Y}\right)}^{D}\exp\left(-d_{ij}^{2}/2\sigma_{Y}^{2}\right)\; & \text{otherwise} \end{array}\right., \end{array}$$

where *Y* ∈ {*E, I*} denotes the population type of pre-synaptic neuron *j*, *D* is the dimensionality of the model space (here *D* = 2, for *D* = 1 see Supplementary Material), and σ_*Y*_ is the spread of the connections of the corresponding type.

We use the terms *configuration* or *profile* to refer to the connection topology of a network. Important parameters that we investigated are σ_*E*_ and σ_*I*_, the connectivity spread of excitatory and inhibitory connections, respectively. We varied both parameters between 5 and 25 pixels, corresponding to 75 and 375 µm in visual cortex scale. These values span a biologically plausible range. For instance, Hellwig (2000) measured a Gaussian decay of connection probability with spreads between 150 and 350 µm for pyramidal neurons in the rat primary visual cortex. Similarly, Mariño et al. (2005) reported a Gaussian connection spread of about 125 µm for local connections in cat V1.

Throughout this study network topologies with the property of Gaussian kernel widths σ_*E*_ < σ_*I*_ are termed **Mexican hat** networks. Likewise, topologies with σ_*E*_ > σ_*I*_ are termed **inverse Mexican hat**. The setting σ_*E*_ = σ_*I*_ is called a **balanced hat**. This naming scheme for topologies featuring two Gaussian kernels can be found throughout the literature, for example in work by Kang et al. (2003); Blumenfeld et al. (2006); Bressloff (2012).

We considered one type of inhibitory and two types of excitatory synapses. Inhibitory synaptic interactions were modeled using instantaneously rising, exponentially decaying functions with a time constant that describes GABA_A_ receptor kinetics. Excitatory synaptic interactions were modeled using the same functions with a time constant that describes AMPA receptor kinetics, and, using a bi-exponential function with larger time constants to describe NMDA receptors. We assumed a fixed ratio between both receptor types with a fraction of 70% AMPA receptors (Myme et al., 2003).

When we relate to the connection strengths, we use the term *operating regime*. Two important parameters in this regard are the maximum conductances ḡ_*EE*_ and ḡ_*IE*_, which quantify the connection strengths between excitatory to excitatory and excitatory to inhibitory synapses. The ratio between maximum AMPA and NMDA was fixed (ḡ_AMPA,*EE*_ = 0.7ḡ_*EE*_ and ḡ_NMDA,*EE*_ = 0.3ḡ_*EE*_, cf. Stimberg et al., 2009). The maximum conductance values were varied in rather small amplitude ranges between 0 and 1.2 nS such that the excitatory post-synaptic potential (EPSP) at resting membrane voltage (-65 mV) had an overall small deflection with a maximum value of up to 1.5 mV. The relation between connection weight and voltage deflection amplitude is visualized in the Supplementary Material. These values are within a biologically plausible range. For instance, Mason et al. (1991) observed single spike-triggered EPSPs ranging from 0.05 up to 2 mV with a mean size of 0.55 mV in rat primary visual cortex. Inhibitory connection strengths were kept fixed (ḡ_*II*_ = ḡ_*EI*_ = 5.0 nS).

Synaptic delays depended linearly on the distance between cells in the model network. We assumed a conduction velocity of 0.2 m/s (about 13,000 px/s) in our networks. Usually values between 0.1 and 0.5 m/s are measured (Bringuier, 1999; González-Burgos et al., 2000).

#### 2.1.2. Network input

Each neuron received afferent inputs modeled as *K*_*A*_ = 100 independent Poisson spike trains targeting AMPA receptors. Hence, inputs to each neuron were decorrelated and all observed correlations could only arise due to recurrent interactions.

We distinguished between a homogeneous, also termed *blank stimulus*, and a heterogeneous tuned input, also termed *orientation stimulus*. In the homogeneous mode, input was not tuned but every neuron received independent Poisson spike trains with the same rate. In the heterogeneous mode the afferent input was tuned according to the preferred orientation of the post-synaptic neuron. The orientation preference of each neuron is determined by its position in the pinwheel map depicted in Figure 1. We assumed moderate orientation tuning. The input firing rate to each cell *i* was computed by:

(2) $$\begin{array}{l} \nu_{\text{Aff},i}\left(s\right)=\left(\nu_{\text{Aff},\text{ max}}-\nu_{\text{Aff},\text{ base}}\right)\exp\left(-\frac{{\left(s-s_{{\text{PO}}_{i}}\right)}^{2}}{2\sigma_{\text{Aff}}^{2}}\right)+\nu_{\text{Aff},\text{ base}}, \end{array}$$

where ν_Aff, max_ is the maximum or peak firing rate, ν_Aff, base_ is the baseline firing, σ_Aff_ is the tuning width, *s* is the orientation of the stimulus (*s* ∈ [−90°, 90°)), and *s*_PO_*i*__ denotes the *i*th neuron's preferred orientation according to the orientation map. Inputs only varied in spatial dimensions and were constant in time.

Network activity was evaluated after the first second of afferent stimulation to avoid artifacts caused by network initializations and onset transients.

#### 2.1.3. Implementation

The network was implemented in Python 2.7 and was partly compiled into C-code for efficiency using the Numba library (The Numba Development Team, 2015). The source code is available online^1^. Some numerical experiments were also conducted with smaller networks of Hodgkin-Huxley type neurons (cf. Stimberg et al., 2009) using the BRIAN simulator package (Goodman and Brette, 2008) (data not shown, example run see Supplementary Video). All data and parameter explorations were managed using the simulation toolkit *pypet* (Meyer et al., 2016).

### 2.2. Data post-processing

#### 2.2.1. Noise correlations

Noise correlations were quantified using the linear correlation coefficient among spike counts. Spike counts *r* were either assessed over repeated fixed length intervals of stimulus presentation or by convolving an observed spike train of a single stimulus presentation with a temporal sliding window kernel *K*_*T*_ (cf. Renart et al., 2010):

(3) $$\begin{array}{l} r_{i}\left(t,T\right)=\int_{t^{\prime }}K_{T}\left(t\prime -t\right)S_{i}\left(t\prime \right) \end{array}$$

where *K*_*T*_ is a sliding window of length *T*, and *S*_*i*_ the spike train of neuron *i* with $S_{i}\left(t\right)=\underset{k}{\sum }\delta \left(t_{i}^{k}-t\right)$. We used the standard expression for the correlation coefficient *r*_*SC*_ of the spike counts:

(4) $$\begin{array}{l} r_{SC}=\frac{\mathrm{Cov}\left(r_{i},r_{j}\right)}{\sqrt{\mathrm{Var}\left(r_{i}\right)\mathrm{Var}\left(r_{j}\right)}} \end{array}$$

where Cov(*r*_*i*_, *r*_*j*_) is the covariance of spike counts between cells *i* and *j* and Var(*r*_*i*_) the individual spike count variance of one cell.

#### 2.2.2. Spatial activity patterns

**Figure 2:**
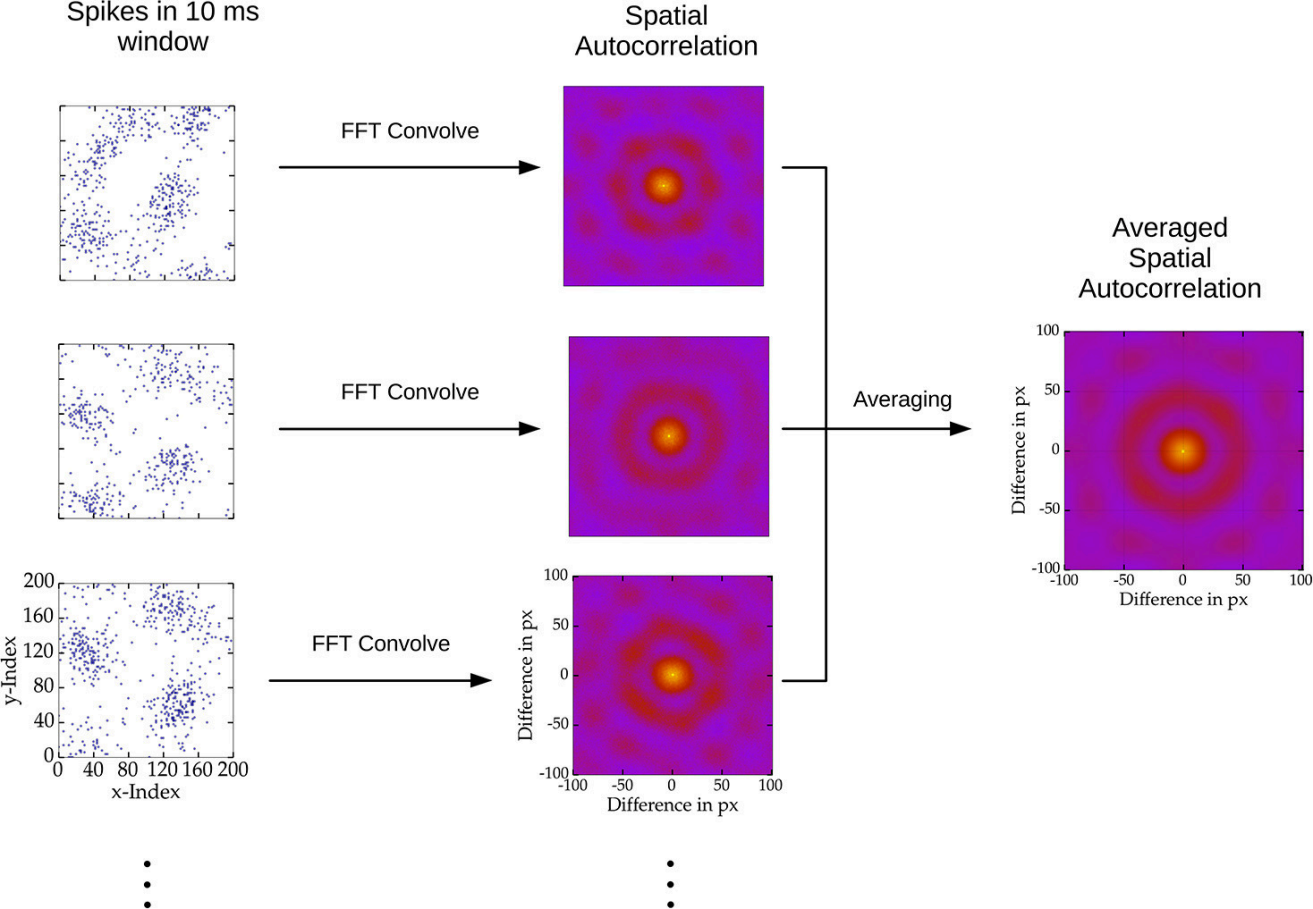
**Depiction of spatial autocorrelation computation**. Spiking activity of 10 ms windows across stimulus presentations were sampled, here depicted as blue dots on the left. Subsequently the spatial autocorrelation of the sampled activity was computed using two dimensional convolution via the fast Fourier transform. Finally, the spatial autocorrelation was average over all samples.

In order to determine the spatial scale of changing patterns we sampled 100 windows of 10 ms activity for each stimulus presentation and counted the spikes in these intervals for each excitatory cell. Accordingly, for each sample interval of activity of the 2D networks, we computed a spatial grid matrix of spike counts. The autocorrelation was calculated convolving the spike count matrix with itself using the fast Fourier method of the scipy package (Jones et al., 2001). Next, to compute the mean spatial autocorrelation of a network we normalized the autocorrelations and averaged across the sampled time windows and stimulus presentations. This procedure is visualized in Figure 2.

#### 2.2.3. Self-sustained activity

If recurrent excitation becomes too strong, i.e., if the excitatory coupling strengths surpass a threshold determined by the strength of lateral inhibition, persistent spiking activity can be observed even in the absence of afferent stimulation. In this case usually spiking activity diverges and the spiking model neurons fire close to their maximum firing rate. In order to test for self-sustained activity, stimulation was applied for 1 s and 1 s after stimulation was terminated^2^, spikes were counted within another 1 s interval. If spikes were observed the network was classified as *self-sustained*, viz. the network was able to sustain spiking without any external drive. For all analyses we only considered activity that was not self-sustained.

#### 2.2.4. Orientation selectivity

In order to quantify the sharpening of network tuning we applied a measure called *Orientation Selectivity Index* (OSI) (Swindale, 1998):

(5) $$\begin{array}{l} \text{OSI}=\sqrt{{\left(\underset{s_{i}}{\sum }\langle r\left(s_{i}\right)\rangle \cos\left(2s_{i}\right)\right)}^{2}+{\left(\underset{s_{i}}{\sum }\langle r\left(s_{K}\right)\rangle \sin\left(2s_{i}\right)\right)}^{2}}\\ /\underset{s_{i}}{\sum }\langle r\left(s_{i}\right)\rangle , \end{array}$$

where 〈*r*(*s*_*i*_)〉 is the average spike count of a model neuron for a particular stimulus *s*_*i*_. We chose twelve evenly spaced orientations *s*_*i*_ ∈ {−90°, −75°, −60°, …, 75°}. 〈*r*(*s*_*i*_)〉 was averaged over 10 stimulus presentations per orientation. The OSI is a measure of tuning sharpness that ranges from 0 to 1. Values close to 0 correspond to weakly tuned neural responses whereas values close to 1 correspond to well tuned activity. We computed the average OSI then across the whole excitatory neuron population in each network.

#### 2.2.5. Fisher information

The quality of a population code was quantified using an estimate of Fisher information. The numerical information experiments were based on repeated presentations of oriented stimuli of −1 and 1 degree. After an initial phase of 1 s, 3 s of stimulation were used to estimate Fisher information. The number of stimulus presentations varied between 125 and 7,000 per oriented stimulus depending on the number of neurons we considered for readout. We chose at least as many stimulus presentations as the number of readout neurons that we sampled. This avoids sets of too few training data items of a number lower than the amount of Fisher estimation parameters (see below). Fisher information was estimated using an approach from Seriès et al. (2004). In most experiments 60% of the simulation data were used to train a locally optimal linear estimator (LOLE) to predict the stimulus orientation from the spiking activity. In the experiments based on 7,000 samples, however, we chose 80% of the stimulus presentations for training. The estimator has the form:

(6) $$\begin{array}{l} ŝ={\text{w}}^{T}\text{r}+w_{0}, \end{array}$$

where ***w*** is a vector of weights, $\text{r}={\left(r_{1},\ldots ,r_{N}\right)}^{T}$ is a vector containing the spike response of the read out neuron sample in the fixed time window of 3 s, and *w*_0_ a bias weight. ***w*** and *w*_0_ were optimized to reduce decoding error based on the given training data. The parameters were fitted via stochastic gradient descent using the scikit-learn Python library (Pedregosa et al., 2011). Training was stopped in case generalization performance on the validation set—containing 20% (10% for 7,000 presentations) of the data—decreased for 500 consecutive epochs. After training was completed, mean and variance of the estimates were computed for both orientations ($s_{1}=-1.0^{{}^{\circ}}$ and $s_{2}=1.0^{{}^{\circ}}$) based on the remaining 20% (10%) of the data. Fisher information was approximated via:

(7) $$\begin{array}{l} I_{\text{LOLE}}=\frac{{\left(\frac{\langle {ŝ}_{2}\rangle -\langle {ŝ}_{1}\rangle }{s_{2}-s_{1}}\right)}^{2}}{\frac{1}{2}\left(\mathrm{Var}\left({ŝ}_{2}\right)+\mathrm{Var}\left({ŝ}_{1}\right)\right)}, \end{array}$$

where 〈ŝ_*i*_〉 denotes expectation and Var(ŝ_*i*_) is the variance of the estimates for one particular stimulus across different presentations. *I*_LOLE_ provides a lower bound on the Fisher information.

We further computed the information available in a data set where all correlations have been artificially removed by shuffling the spike counts of individual neurons observed across different stimulus presentations (*I*_shuff_). An example of how data is shuffled across stimulus presentations is given in Table 1. A comparison between *I*_LOLE_ and *I*_shuff_ shows whether correlations improve (*I*_LOLE_ > *I*_shuff_), degrade (*I*_LOLE_ < *I*_shuff_) or have no effect (*I*_LOLE_ = *I*_Shuff_) on stimulus encoding quality. Additionally, we computed the diagonal information *I*_diag_ to investigate whether the correlations themselves carry information about the stimulus. To estimate *I*_diag_ the decoder was trained on the shuffled data but applied to the original one. In case *I*_diag_ is smaller than *I*_LOLE_, correlations carry information that would be lost if the decoder ignores the correlation structure. Note that the value of *I*_diag_ is bounded by *I*_LOLE_ from above.

**Table 1:** **These tables give an example of shuffled data that can be used to compute the shuffled information ***I***shuff**.

**Neuron**	**Trial 1**	**Trial 2**	**Trial 3**	**Trial 4**	**Trial 5**	**Total**
Neuron I	1	2	3	4	5	15
Neuron II	1	1	0	3	3	8
Neuron III	1	2	0	5	4	12
**Neuron**	**Shuff 1**	**Shuff 2**	**Shuff 3**	**Shuff 4**	**Shuff 5**	**Total**
Neuron I	3	4	1	5	2	15
Neuron II	3	0	1	1	3	8
Neuron III	4	1	5	2	0	12

*The top table lists the original spike counts observed for 3 hypothetical neurons over 5 trials. The bottom table depicts the shuffling of trials. The observed spike counts are shuffled for each neuron. Note that counts are shuffled across trials but not across neurons. Hence, the totally observed number of spikes for each neuron (last column) remains equal between the non-shuffled and shuffled condition*.

Moreover, in order to test if the linear decoder is suitable for information estimation, we used a non-linear support vector regression (SVR) decoder with radial basis function (RBF) kernels for comparison. We transformed the observed spike count data to zero mean and variance one, and optimized the two SVR hyperparameters by grid search. We explored the penalty function parameter of the SVR error (*C*_*SVR*_ ∈ {0.01, 0.1, 1, 10}) as well as the width of the RBF kernel (γ_*SVR*_ ∈ {0.0001, 0.001, 0.01, 0.1}) using 5-fold cross-validation on the training set before validation on the test data.

For all information processing analyses we only considered excitatory neurons because GABAergic inhibitory interneurons are not known to project out of primary visual cortex (Seriès et al., 2004; Schmolesky, 2007).

## 3. Results

We tested network models of different sizes using either 100 × 100 or 200 × 200 excitatory neurons with 2,500 or 10,000 inhibitory neurons, respectively. These two sizes correspond to 1.5 mm × 1.5 mm and 3 mm × 3 mm of cortical area. For the smaller (100 × 100) networks we also simulated responses to heterogeneous, orientation tuned input using an orientation map consisting of 4 pinwheels as depicted in Figure 1. The smaller networks were also used for the assessment of the quality of stimulus encoding.

### 3.1. Correlated variability for homogeneous external input

**Figure 3:**
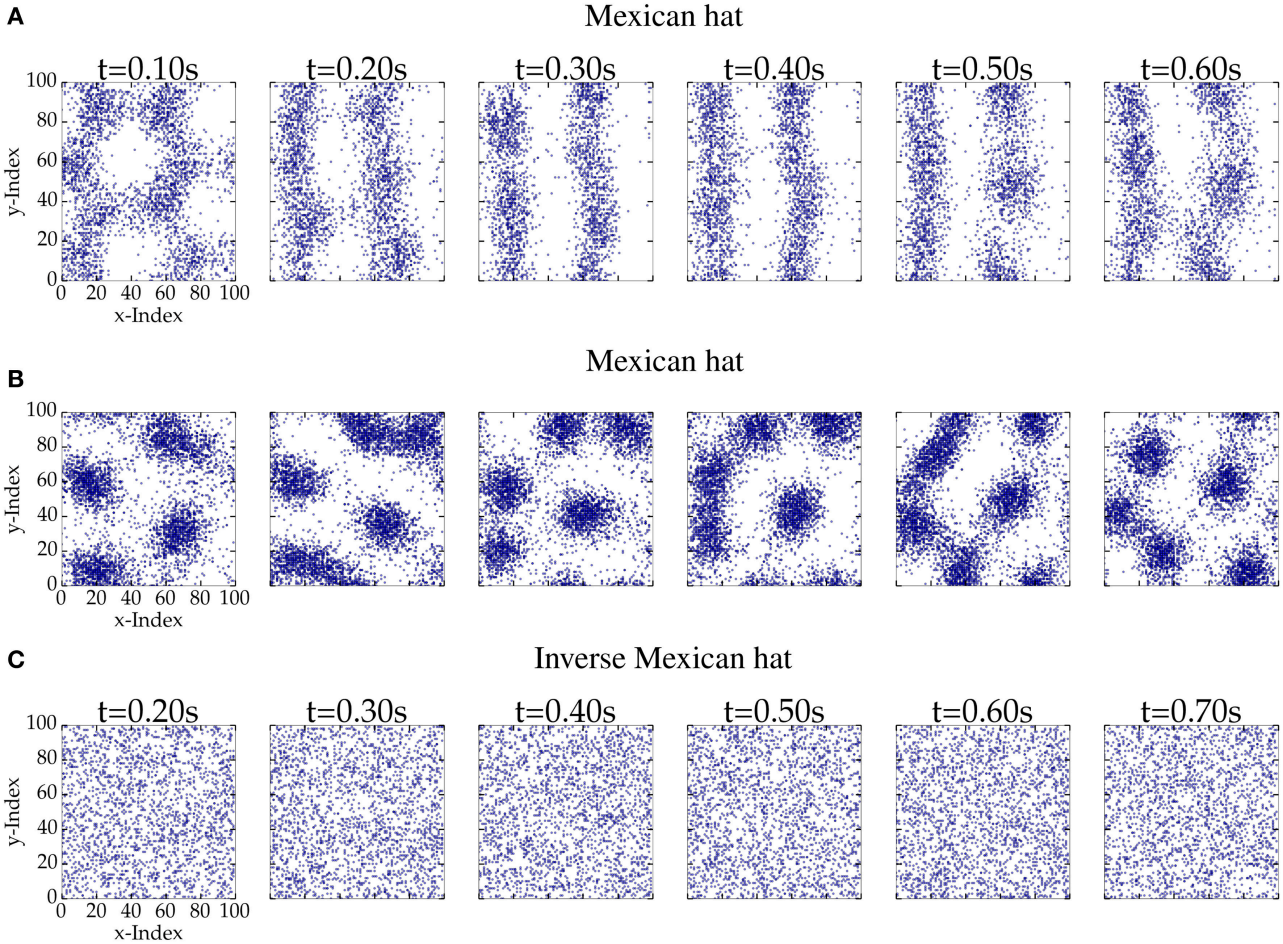
**Consecutive snapshots of 100 ms length of network activity for networks of 100 px × 100 px**. A blue dot corresponds to an excitatory spike within the time interval. The top (σ_*E*_ = 10 px < σ_*I*_ = 15 px, ḡ_*EE*_ = 0.4 nS and ḡ_*IE*_ = 0.6 nS) and the middle row (ḡ_*EE*_ = 0.32 nS and ḡ_*IE*_ = 0.4 nS) provide the activity pattern of a Mexican hat network. The bottom row (σ_*E*_ = 15 px > σ_*I*_ = 10 px, ḡ_*EE*_ = 0.4 nS, and ḡ_*IE*_ = 0.6 nS) shows activity of an inverse Mexican hat network. **(A)** Mexican hat; **(B)** Mexican hat; **(C)** Inverse Mexican hat.

First, we investigated the firing patterns that emerge for different network configurations. Figure 3 shows consecutive snapshots of spiking activity for different network parameterizations: Two Mexican hat and one inverse Mexican examples. The spiking activity of Mexican hat networks revealed bump or stripe like patterns. Bumps may move and fuse together, but also eventually appear, disappear, pulsate or turn into flickering stripes. These stripes can be stable over a few seconds and may turn back into circular bumps for the same network realization. In contrast, for inverse Mexican hat (Figure 3C) and balanced networks (data not shown) we did not observe spatially inhomogeneous patterns.

**Figure 4:**
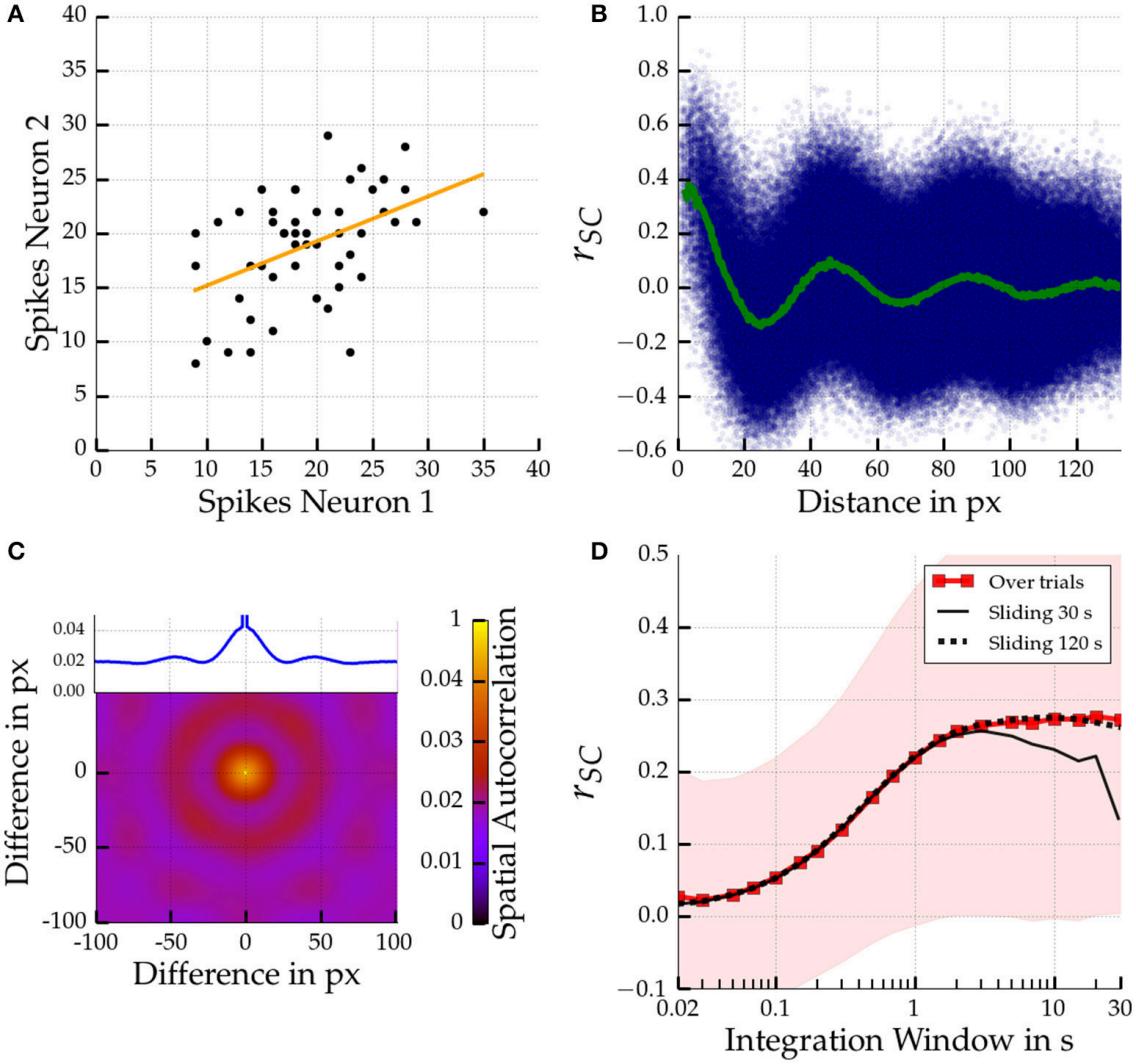
**Spatial scales of noise correlations for a large 200 px × 200 px Mexican hat network**. Parameters are σ_*E*_ = 10 px < σ_*I*_ = 15 px, ḡ_*EE*_ = 0.4 nS, ḡ_*IE*_ = 0.6 nS. Top left **(A)**: Distribution of spike counts given a 10 s integration time window for two pairs of two directly neighboring cells. The corresponding correlation coefficient is *r*_*SC*_ = 0.46 (*p* < 0.001). The regression line is given in orange. Top right **(B)**: Noise correlations for pairs of cells according to distance computed across 50 stimulus presentations using a 1 s integration window. The correlation coefficient of each individual cell pair is indicated by a small blue dot. The average coefficient of correlation as a function of distance is shown in green. A decaying oscillation of average correlations can be observed. Bottom left **(C)**: Spatial Autocorrelation of the spike counts in the Mexican hat network. Inset shows autocorrelation along the horizontal axis. Bottom right **(D)**: Temporal scales in terms of the average spike count correlation coefficient as a function of integration window size averaged across all cells at most 13.3 px (which corresponds to 0.2 mm in the scaling to cat cortex) apart. The thin black lines show the average noise correlation if estimated from sliding windows over a single experimental run of 30 s and 120 s (dotted). The envelope shows the standard deviation of noise correlation among all cell pairs.

We next quantified the noise correlations for different network settings. We computed the spike count correlation coefficient among pairs of cells for repeated stimulus presentations. An example distribution of spike counts for a pair of neighboring cells (1 px apart) is shown in Figure 4A. Figure 4B displays the distribution of spike count correlation coefficients among different cell pairs for a large 200 × 200 Mexican hat network as a function of distance. The average correlation as a function of distance is denoted by the green line and follows a damped wave pattern. We did not make such an observation for balanced or inverse networks (data not shown). The decay of the average correlation (green line) happened rather quickly and there was a considerable decrease in the amplitude within the first 100 pixels, which corresponds to 1.5 mm in visual cortex (see Section 2). The oscillation frequency of the average *r*_*SC*_ was 2.2 cycles per 100 pixels.

We hypothesized that noise correlations are caused by time-varying, spatially inhomogeneous activity patterns, such as moving bumps. We, therefore, investigated whether the spatial scale of noise correlations corresponds to the spatial scale of the moving bumps. Indeed, the spatial frequency of the noise correlations matches the spatial autocorrelation of the heterogeneous activity profile as depicted in Figure 4C. Both are on the order of 2.2 cycles per 100 pixels. We made similar observations for one-dimensional networks where the relation between spatial frequency and bump size becomes even more obvious because one can easily count the number of bumps emerging in a network (see Supplementary Material).

Experimental studies reported a saturation of spike count noise correlations for increasing time windows (Bair et al., 2001; Reich, 2001; Smith and Kohn, 2008). We were interested if this phenomenon can be observed in our model as well and therefore investigated the temporal scale of the noise correlations. Figure 4D shows the average correlation among cell pairs with a maximum distance of 13.3 px, corresponding to 0.2 mm in visual cortex, for the large Mexican hat network. We found a saturation of the magnitude of the distance dependent correlations when increasing the integration time window. The magnitude saturates for time windows of about 1 s and longer. Similar observations could be made for the calculation of noise correlations with a sliding window and a long single stimulus presentation of 120 s (dotted black line). Usage of a shorter stimulus presentation of 30 s yielded similar results but only up to sliding windows with a length of about 3 s (black line).

**Figure 5:**
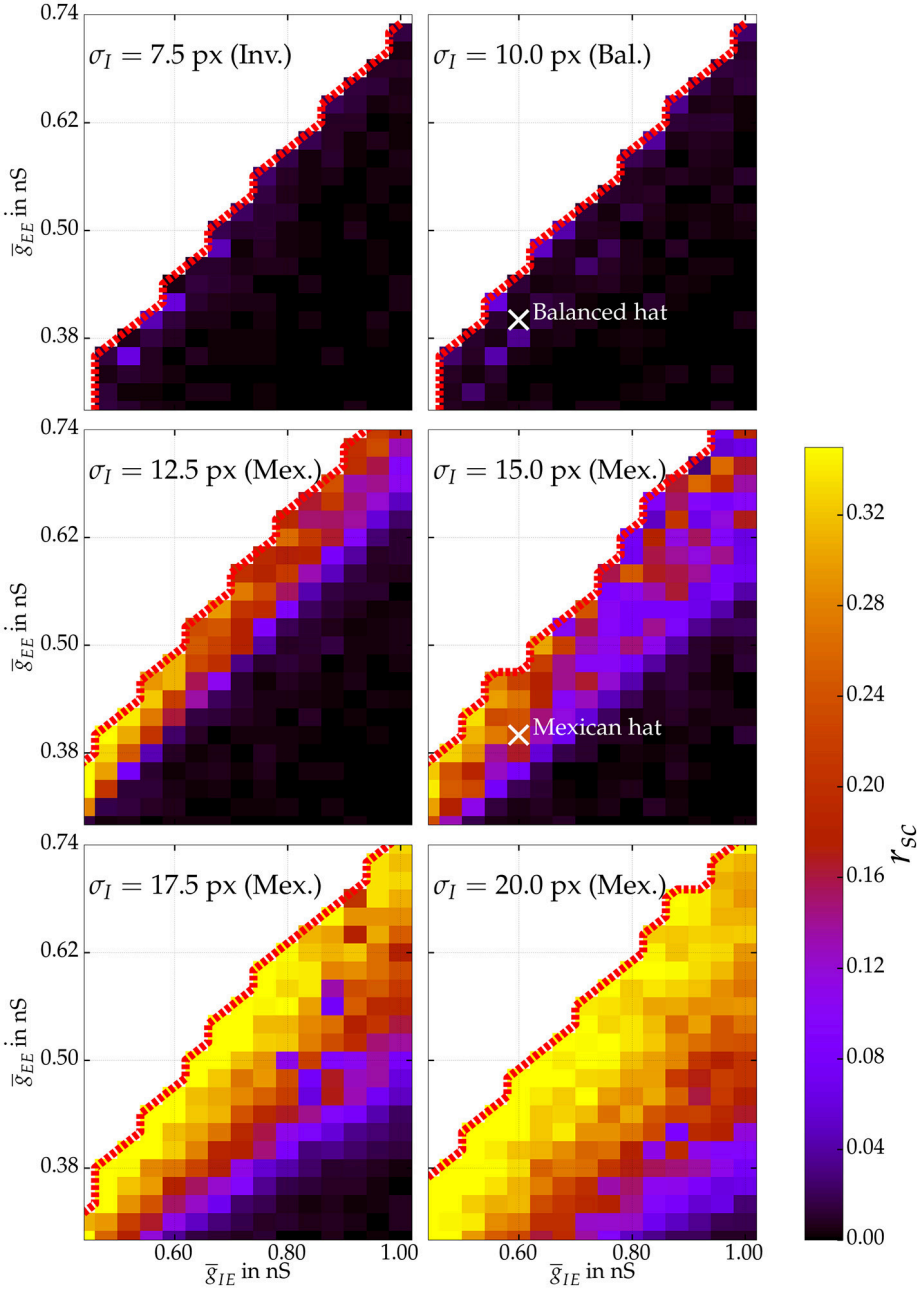
**Average noise correlations of cell pairs at most 13.3 px apart in networks with 100 × 100 excitatory neurons**. The coefficient is estimated using a sliding window of 1 s over one stimulus presentation of 30 s for various network topologies exploring excitatory synaptic coupling strengths ḡ_*EE*_ and ḡ_*IE*_. The thick dotted red line marks the bifurcation to self-sustained activity. Excitatory spread σ_*E*_ is fixed to 10 px. Hence, the top row shows correlations for an inverse and balanced spread, whereas below correlations for Mexican hat networks are shown. White crosses mark the parameter settings of balanced and Mexican hat networks that have also been used for estimating information processing quality in Section 4.2.

Rosenbaum and Doiron (2014) demonstrated that neural network activity can be balanced for Mexican hat configurations due to finite size effects. Accordingly, we were interested if we could observe balanced and uncorrelated neural responses for Mexican hat networks and determine the parameter regimes for which correlations emerge. We varied the parameters ḡ_*EE*_ and ḡ_*IE*_ of the connection strengths for several networks with 100 × 100 excitatory neurons. Figure 5 depicts the average noise correlation among cell pairs at most 13.3 px apart as a function of different recurrent connection strengths for different network configurations. To reduce simulation time the noise correlations were calculated based on a stimulus presentation of 30 s and using a sliding window of 1 s. Noise correlations were observed for Mexican hat configurations with recurrent connectivity strengths close to the boundary of self-sustained activity (red dotted lines). For weak recurrent connection strengths no correlations were observed, indicating a dominant finite size effect. Increasing the width of the Mexican hat, i.e., scaling σ_*I*_ while σ_*E*_ was fixed, we measured strong correlations for a much larger range of parameter values, i.e., these networks are less affected by finite size effects.

Moreover, we investigated the relation between network size and correlations directly in one-dimensional networks (see Supplementary Material). We could show that increasing the number of neurons while keeping all other parameters fixed (including the extent of the network) increased noise correlations in Mexican hat networks.

### 3.2. Correlated variability for orientation stimuli

**Figure 6:**
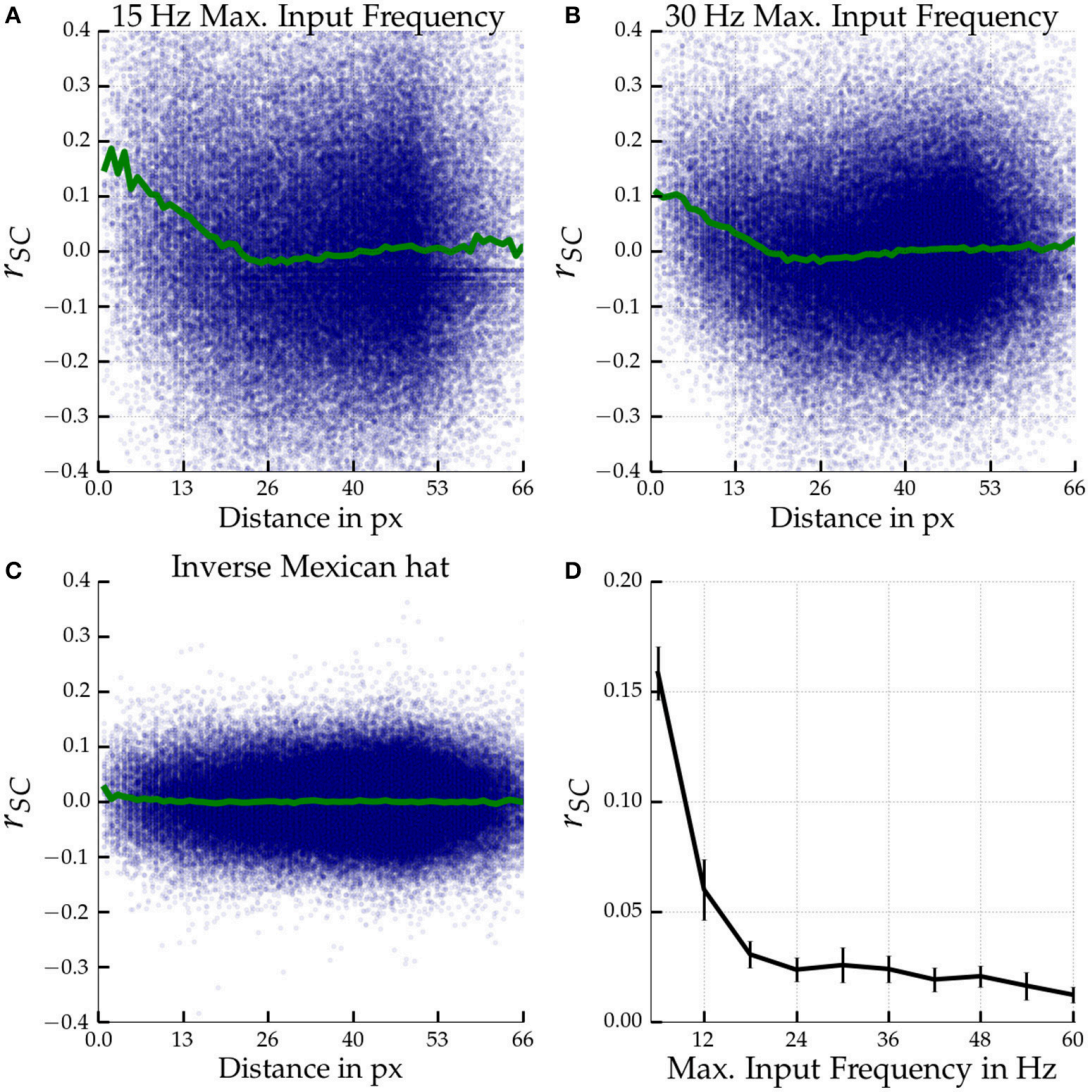
**Noise correlations for tuned input**. Top row **(A,B)**: Noise correlation as a function of distance in a Mexican hat network (σ_*E*_ = 10 px < σ_*I*_ = 15 px) close to self-sustained activity (ḡ_*EE*_ = 0.4 nS, ḡ_*IE*_ = 0.6 nS) driven by tuned input. Bottom left **(C)** shows results for an inverse Mexican hat (σ_*E*_ = 15 px > σ_*I*_ = 10 px) with the same recurrent coupling strengths. The maximum input frequencies were ν_Aff, max_ = 15 Hz **(A,C)** and 30 Hz **(B)**. The blue dots indicate the correlation coefficients measured for individual neuron pairs according to the distance between the two model neurons. The green curve depicts the coefficient averaged across pairs. Bottom right **(D)**: Noise correlations of cells at most 13.3 px apart in Mexican hat networks operating close to self-sustained activity (σ_*E*_ = 10 px < σ_*I*_ = 15 px, ḡ_*EE*_ = 0.4 nS, ḡ_*IE*_ = 0.6 nS) are shown as a function of maximum input frequency ν_Aff, max_. Errorbars mark standard deviations over 5 network realizations. Correlation coefficients were averaged across 6 stimulus orientations (−89°, −59°, −29°, 1°, 31°, 61°) with 30 presentations per orientation and 1 s per trail.

Figures 6A,B show the distance dependence of noise correlations in a Mexican hat network close to self-sustained activity for orientationally afferent external drive of different maximum strengths. Noise correlations decrease linearly with distance within the first 25 pixels, corresponding to about 0.4 mm of visual cortex. The strong oscillatory fluctuations of noise correlations that we observed for homogeneous stimuli almost vanished and we observed only a minor dip below zero of the average noise correlation as a function of distance. As expected, no distance dependent correlations were observed for an inverse Mexican hat network with the same recurrent coupling strengths (Figure 6C). Overall the magnitude of noise correlations decreased for Mexican hat networks in comparison to the previous experiments with a blank stimulus. Increasing the maximum input rate ν_Aff, max_ from 15 Hz (Figure 6A) to 30 Hz (Figure 6B) slightly decreased the magnitude of the noise correlations further. We systematically investigated the influence of the maximum rate ν_Aff, max_ of the tuned input on correlated variability. Figure 6D shows that the magnitude of noise correlations decreases with an increase in maximum rates.

**Figure 7:**
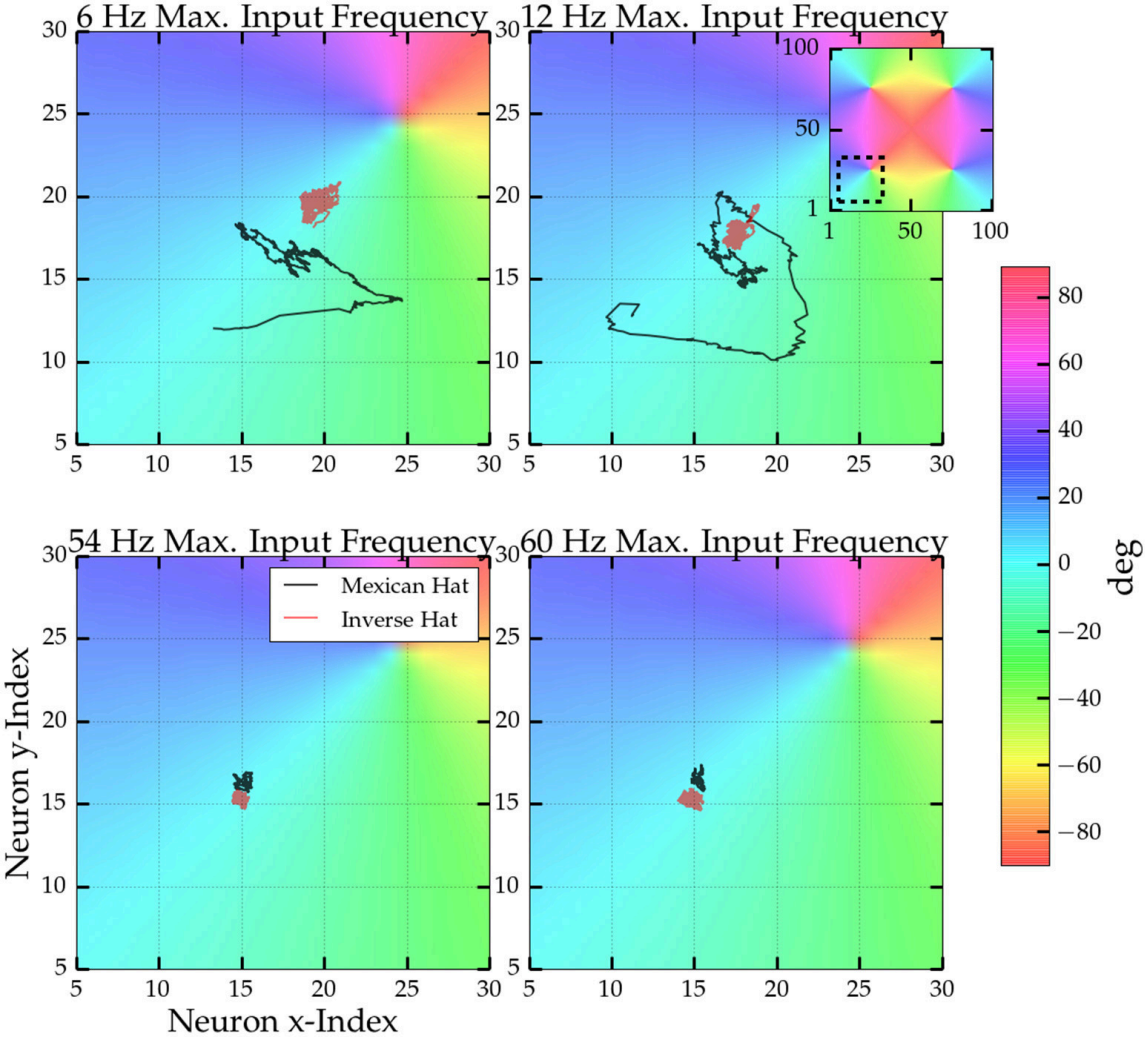
**Trajectories of the center of activity at one pinwheel for four different maxium driving frequencies**. Colors indicate the orientation preferences of the model neurons. The inset shows which part of the full orientation map is depicted. Centers were computed from the activity within a sliding window of 250 ms length. Every neuron's position was interpreted as a vector in the 2D plane and the trajectory is the average vector weighted by the neurons activity within the sliding window. Black line: Trajectories for a Mexican hat network (parameters as in Figure 6) and a stimulus oreintation of 1°. Red lines: Trajectories for an inverse Mexican hat network (σ_*E*_ = 15 px > σ_*I*_ = 10 px) with the same connections strengths and a stimulus orientation of 0°.

Next, we were interested if—similar to the results obtained for homogeneous input before—the noise correlations observed for orientationally tuned input can be linked to movement of the bump patterns. For tuned input the network activity was high within four particular regions of the network (see Supplementary Video), where the preferred orientation of the model neurons correspond to the orientation of the driving stimulus in the orientation map with 4 pinwheels. We examined the movement of the centers of these activity clusters over time. In Figure 7 four trajectories of the activity center (black lines) at one of the network's pinwheels is depicted for an afferent stimulus with an orientation of 1 degree. For low maximum firing rate of the afferent input we identified a considerable amount of movement in the plane, but increasing the input rate significantly reduced movement. In comparison, the red lines show the same trajectories for an inverse Mexican hat driven by a stimulus of 0 degree. There, the center of activity was stable even for low maximum input rates.

### 3.3. Information processing

In this section we demonstrate how the correlations in the network model influence stimulus encoding. We measured and quantified the effects of correlations on coding quality in terms of the three information measures introduced in Section 2: Using Fisher information, shuffled information, and diagonal information.

#### 3.3.1. Fisher information and tuning

We compared Fisher information between networks with a Mexican hat configuration, a balanced configuration (see also white crosses in Figure 5), and an inverse Mexican hat profile.

**Figure 8:**
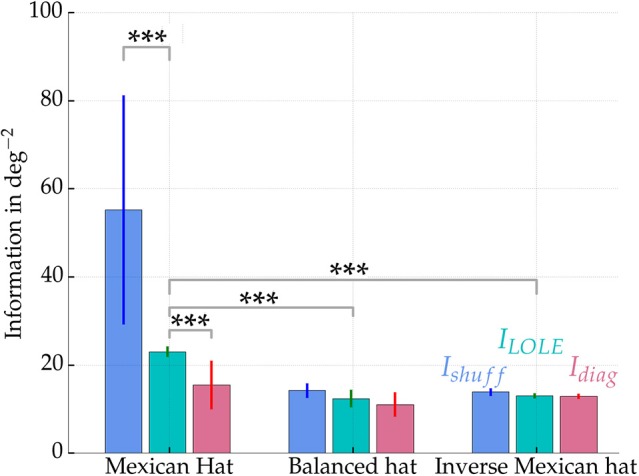
**Fisher information measures ***I***shuff, ***I***LOLE, and ***I***diag for networks with Mexican hat, balanced and inverse Mexican hat connection schemes**. Values were computed from sampling 500 of the 10,000 excitatory neurons and averaged over 10 networks with different recurrent connectivity realizations. Error bars show standard deviations over 10 network realizations. A maximum afferent firing rate of ν_Aff, max_ = 15 Hz was used. Parameters were for Mexican hat σ_*E*_ = 10 px < σ_*I*_ = 15 px, balanced σ_*E*_ = 10 px = σ_*I*_ = 10 px and inverse Mexican hat σ_*E*_ = 15 px > σ_*I*_ = 10 px. Strengths were chosen equally for all networks with ḡ_*IE*_ = 0.6 nS and ḡ_*EE*_ = 0.4 nS (see also white marks in Figure 5). Information was estimated using an approach by Seriès et al. (2004). Significance values are based on a non-parametric Wilcoxon rank-sum test for comparisons of *I*_LOLE_ between different topologies (*p* < 0.001, 10 networks samples) and a Wilcoxon signed-rank test (*p* < 0.001, 10 network samples) for comparisons within the Mexican hat configuration. ^***^p < 0.001

Figure 8 summarizes the information measures for all three network configurations estimated from 500 excitatory readout neurons. The noise correlations—emerging within the Mexican hat network—significantly reduced the stimulus encoding quality in terms of reducing the Fisher information in comparison to the shuffled data (blue and cyan bars in Figure 8 on the left, Wilcoxon signed-rank test, *p* < 0.001, 10 network samples). Furthermore, the diagonal information *I*_diag_ (left magenta bar) is significantly smaller than *I*_LOLE_ (left cyan bar) (Wilcoxon signed-rank test, *p* < 0.001, 10 network samples). Thus, the correlations themselves carried information, and a decoder cannot safely ignore correlated variability without facing a penalty in performance. Interestingly, the total information that could be recovered from the Mexican hat network was significantly larger than in the other two networks (*I*_LOLE_, cyan bars) despite the presence of noise correlations (Wilcoxon rank sum test, all *p* < 0.001, 10 network samples each).

This increase in Fisher information can be explained by the sharpening of orientation tuning by Mexican hat type connectivity patterns, which enhance stimulus encoding of low dimensional stimuli (Zhang and Sejnowski, 1999; Dayan and Abbott, 2005). Thus, despite the presence of correlations, a Mexican hat profile may better encode the stimulus by sharpening of responses. The response of the individual neurons is more selective to orientation stimuli and thereby facilitate discrimination between stimuli.

**Figure 9:**
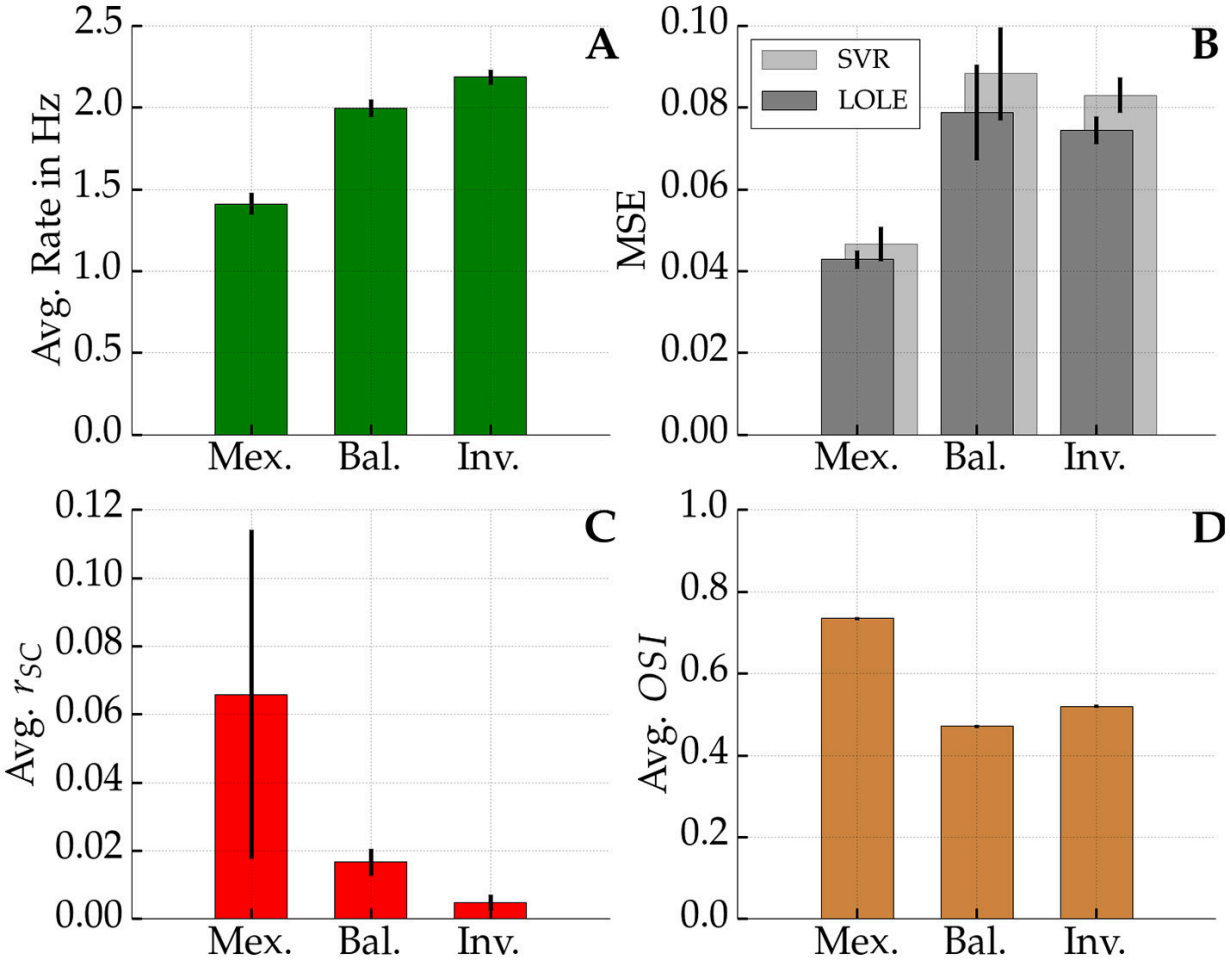
**Firing rate, MSE, ***r***_***SC***_, and OSI**. Top left **(A)**: Average network firing rate averaged over all excitatory neurons. Top right **(B)**: In dark gray the average mean squared error (MSE) of the linear decoder is shown whereas in light gray one sees the average MSE of the best Support Vector Regression (SVR). Bottom left **(C)**: Average noise correlation among pairs of neurons at most 13.3 px apart for a 3 s time window. Bottom right **(D)**: Average Orientation Selectivity Index (OSI) among all neurons in the networks. Black bars show the standard deviation across 10 network realizations per profile in each sub-figure. In **(D)** standard deviations are so low that the black bars are not visible. Network parameters as in Figure 8.

In order to quantify the sharpening of tuning we applied the *Orientation Selectivity Index* (OSI) measure (see Equation 5). Figure 9D shows the average OSI of the different networks. The Mexican hat networks exhibited the sharpest tuning (0.734 ± 0.003). The inverse configuration showed the second sharpest tuning (0.520 ± 0.003) followed by the balanced network with (0.471 ± 0.003). All these differences are significant (Wilcoxon rank-sum test, all *p* < 0.001, 10 sample networks each). Notably, the coding was not simply improved due to the availability of more spikes. In Figure 9A it can be seen that the average firing rate of the network was smaller for Mexican hat networks. Sharper tuning implies that on average fewer neurons respond to an input stimulus, which in turn decreases the average network firing rate. In summary, despite stronger noise correlations for Mexican hat networks (Figure 9C), the coding quality improves alongside a sparser spiking code.

We further tested if the decoding performance could be improved over LOLE by using non-linear SVR. However, there was no increase in performance. On average its mean squared error (MSE) was slightly worse than the error of the linear decoder, as shown in Figure 9B. This suggests that the linear decoder is close to optimality and it is unlikely that there is information that could only be obtained by using non-linear methods.

#### 3.3.2. Fisher information and lateral connectivity

**Figure 10:**
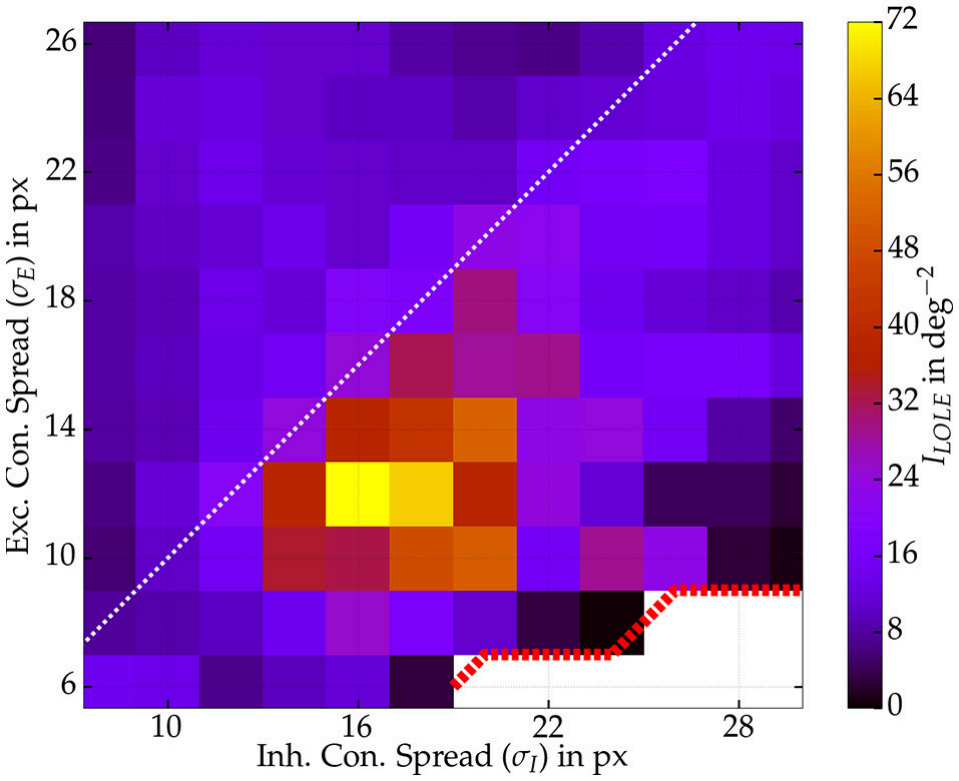
**Fisher information ***I***LOLE as a function of both connection widths σ***I*** and σ***E*****. One network per spread combination with recurrent strengths of ḡ_*EE*_ = 0.4 nS and ḡ_*IE*_ = 0.6 nS. Some Mexican hat networks with very narrow σ_*E*_ led to self-sustained activity (white area, red dotted border) and were excluded from the analysis. Values were computed from 125 excitatory neurons and 125 repetitions per stimulus (*s* ∈ {−1°, 1°}). Maximum input firing rate was 30 Hz.

We tested whether the performance gain is indeed caused by the Mexican hat configuration or rather an effect of the width of the inhibitory connection spread only. Therefore, we explored different widths of both connection spreads (σ_*I*_ and σ_*E*_) and kept recurrent coupling strengths fixed (ḡ_*EE*_ and ḡ_*IE*_). To reduce simulation time we ran 125 repeated presentations per input stimulus to estimate Fisher information. We sampled activity from 125 excitatory readout neurons. Clearly, as depicted in Figure 10, the phenomenon is related to the Mexican hat configuration. Best stimulus encoding performance was achieved by network topologies with shorter excitatory than inhibitory connection spread (σ_*E*_ < σ_*I*_). Still, benefits in information processing are limited to a range of Mexican hats. As the area on the right side of the image shows. A very wide spread of inhibitory connections, however, led to a drop of Fisher information also for Mexican hat topologies.

#### 3.3.3. Fisher information and sample size

**Figure 11:**
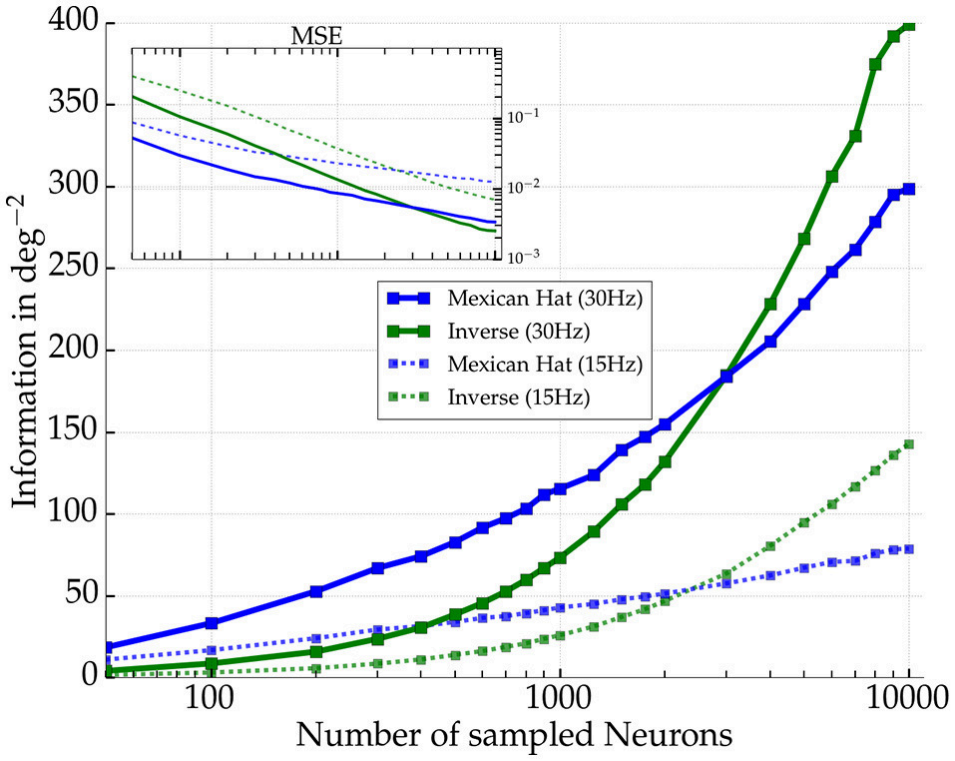
**Amount of Fisher information as a function of number of excitatory neurons sampled for two network realizations**. Mexican hat (σ_*E*_ = 10 px < σ_*I*_ = 15 px) in blue and inverse Mexican hat (σ_*E*_ = 15 px > σ_*I*_ = 10 px) in green (both with ḡ_*IE*_ = 0.6 nS and ḡ_*EE*_ = 0.4 nS). Solid (dashed) lines indicate results for a maximum afferent rate of 30 Hz (15 Hz). The inset shows the corresponding mean squared error (MSE) of the LOLE decoder.

We further wanted to know how the difference in Fisher information between the network types depends on sampling of the number of readout neurons for Mexican hat and the inverse Mexican hat networks (cf. Averbeck et al., 2006). We repeated the previous experiments with the same parameter settings but used 7,000 presentations per stimulus and estimated *I*_LOLE_ again from the spike counts of different numbers of excitatory readout neurons. Figure 11 summarizes the results. The better performance was observed for Mexican hat networks as long as roughly fewer than one third of the neurons were included. However, if more readout neurons were used to reconstruct the stimulus, *I*_LOLE_ was higher for the inverse Mexican hat networks. Hence, for larger samples of neurons, the detrimental nature of the noise correlations was more pronounced and eradicated the advantage of sharper tuning in Mexican hat networks.

## 4. Discussion

### 4.1. Dynamics and noise correlations

#### 4.1.1. Theoretical considerations

We demonstrated that recurrent connectivity can cause correlated variability. As we hypothesized, in networks of adaptive exponential integrate and fire neurons, Mexican hat coupling with wider inhibitory than excitatory connectivity spread leads to noise correlations.

Our findings are in line with analytical results from Rosenbaum and Doiron (2014). The authors used a rate based model network to investigate how recurrent topologies in 1D and 2D networks affect excitatory and inhibitory recurrent inputs. Inverse Mexican hat configurations and those with an equally wide spread of excitatory and inhibitory connections yield a stable balance of currents, i.e., excitatory and inhibitory recurrent currents were of similar magnitude and canceled each other. This leads to constant stable firing rates of all neurons. We could show in our simulations that this stable balance further leads to a decorrelation of network activity. Rosenbaum and Doiron (2014) derived that Mexican hat coupling cannot maintain a stable balance between recurrent excitation and inhibition, thereby amplifying spatial frequencies in the network activity. The authors further demonstrated that the network size also influences this amplification. If the network size is very small or recurrent connectivity is very weak, Mexican hat networks may still exhibit homogeneous firing activity. Likewise, we observed homogeneous responses for operating regimes far from the region of self-sustained activity (see Figure 5). We could also demonstrate (see Supplementary Material) that increasing network size can lead to noise correlations in Mexican hat networks operating in regions afar from self-sustained activity.

An analytical solution of the network state could not be provided for the case that spatial frequencies are amplified by Gaussian Mexican hat connectivity (Rosenbaum and Doiron, 2014). Nevertheless, for simplified connection topologies that also feature wider inhibitory than excitatory connection spread, it has been shown that networks give rise to spatially inhomogeneous responses (Pinto and Ermentrout, 2001; Coombes, 2005; Guo and Chow, 2005; Roxin et al., 2006). For example, Hansel and Sompolinsky (1998) showed that using a sinusoidal spatial connectivity pattern the network undergoes a Turing bifurcation if the recurrent strength of the sinusoidal component reaches a critical value. The resulting network state is given by a single bump solution. Moreover, the authors demonstrated that the spatially inhomogeneous activity starts moving across the spatial extent of the network in case neuronal adaptation is considered in the rate based network model. This movement then leads to joint modulations of firing rates and, consequently, to correlation among rates. This is also sketched in the animation provided in the Supplementary Material. In our simulations of spiking neurons joint modulations in firing rates manifested themselves in joint modulations of spiking activity which appear as dynamic patterns on a network level, i.e., bumps and stripes. Due to the joint changes of spiking activity we measured distance dependent noise correlations. The pairwise correlations were modulated in a damped oscillatory manner. These phenomena, i.e., the pattern formation and noise correlations, could be observed for a wide range of parameter settings and were most strongly pronounced close to the boundary to self-sustained activity.

The locations of emerging activity bumps can be determined and steered by inhomogeneous inputs Bressloff (2012). We made a similar observation for our networks using an inhomogeneous Poisson input that simulates an oriented bar stimulus. Inhomogeneous input locked bump activity at the locations corresponding to the peaks of the afferent input, but a weak drift or jitter of the activity could be observed as well (see Figure 7). Noise correlations persisted for these heterogeneous inputs, but were reduced in comparison to homogeneous inputs. The activity patterns and spatial dependency of correlations were also modulated in comparison to homogeneous inputs. We observed an almost linear decay with distance (Figures 6A,B). In contrast to the homogeneous input scenario, the average *r*_*SC*_ did not reach values considerably below 0. In addition, noise correlations as well as the movements or jitter around the peak locations were stronger for weak external drive.

#### 4.1.2. Comparison to other modeling studies

Keane and Gong (2015) hypothesized that phenomena such as neuronal spiking variability and noise correlations may be explained by macroscopic network dynamics such as traveling waves. The authors simulated a two-dimensional spiking neural network model where the connection probability did not depend on distance between cells but the coupling strengths of excitatory neurons did. The coupling strengths among excitatory neurons exhibited a Gaussian profile similar to our connectivity kernel. Inhibitory neurons, however, were coupled uniformly. Afferent inputs were either noisy or deterministic and homogeneous. The authors observed moving bumps activity as well as traveling wave fronts and noise correlations following a damped sine wave as a function of cell pair distance. They compared their results to topologies with uniform random connections where no pattern formation and noise correlations were observed. In our models connection strengths were constant across all synapses but connection probabilities depended on distance. In comparison to their study, we also applied Gaussian kernels to inhibitory connections, did extensive parameter explorations to uncover the influence of kernel sizes and connection strengths on correlations, and investigated inhomogeneous inputs and the relation between noise correlations and information.

Yger et al. (2011) researched the influence of Gaussian topology in terms of inhibitory and excitatory connection width on noise correlations. Surprisingly, they observed the emergence of bump patterns and noise correlations only for very narrow excitatory spreads (σ_*E*_ ≪ σ_*I*_). Yet, they kept recurrent weights fixed and used a rather low coupling strength. Therefore, it is likely that the network size was too small for the given recurrent weights and the finite size effect kept network activity stable.

Ponce-Alvarez et al. (2013) used a one-dimensional ring network of non-linear rate model neurons with different preferred stimulus orientations to study phenomena observed in the area MT of awake monkeys. The rate units were connected via a Mexican hat like sinusoidal coupling. The authors hypothesized that this network topology can explain the experimentally observed directional tuning of noise correlations in MT neurons. For particular parameter regimes with strong weight on the sinusoidal component of the coupling configuration Ponce-Alvarez et al. (2013) observed bump patterns similar to ours.

Moreover, Rosenbaum et al. (2017) provide an alternative hypothesis for the emergence of noise correlations. While we solely focused on recurrent coupling, they investigated the interplay of recurrent and afferent feed-forward connections. In contrast to our model where each neuron simply received independent Poisson input, in Rosenbaum et al. (2017) afferent inputs could be shared among post-synaptic cells, depended on the distance among neurons, and afferent connection probabilities followed a spatial Gaussian kernel (with width σ_*A*_) similar to our recurrent coupling. Using simulations of spiking neurons as well as mean-field approximations, Rosenbaum et al. (2017) showed that if afferent connection spread is narrower than excitatory or inhibitory recurrent connections (σ_*A*_ < σ_*E*_, σ_*I*_), distance dependent noise correlations similar to our results emerge.

#### 4.1.3. Biological relevance of the results

In our study we showed that noise correlations can emerge by moving patterns of higher and lower spiking activity. On the single cell level phases of lower spiking activity alternate with phases of higher activity, depending on whether the neuron is part of a more or a less active network region.

Mochol et al. (2015) showed in the auditory cortex of anesthetized rats that noise correlations are dominated by phases of coinactivation. During spontaneous activity, the authors observed periods where all neurons in a local network jointly reduced firing. This resembles the occurrence of bump activity states in our networks where firing rates of neighboring model neurons were jointly reduced if they are part of an off region of a bump. Moreover, Mochol et al. (2015) demonstrated that external stimulation led to a reduction of these low activity periods which went hand in hand with a decrease in the magnitude of noise correlations. Similarly, our simulation showed that increasing the strength of an orientation tuned stimulus led to a decrease of correlated variability in a 2D network (see Figure 6D).

Moreover, increasing input strength can be a suitable model of an increase of stimulus contrasts in biological experiments (Rubin et al., 2015). Kohn and Smith (2005) reported weaker correlations for strong contrasts of drifting orientation gratings in experiments with Macaque monkeys. These findings match our simulations with increasing afferent input of inhomogeneous stimuli.

In our simulations noise correlations depended on the size of the integration time window (Figure 4D). We observed an amplitude saturation for large windows. This is in line with findings from biological experiments (Bair et al., 2001; Reich, 2001; Smith and Kohn, 2008).

Smith and Kohn (2008) and Rosenbaum et al. (2017) measured correlations in visual cortex of monkeys alongside the neural tissue using a micro-electrode array (MEA) and drifting sinusoidal grating stimuli. Both reported a distance dependence of noise correlations. However, the spatial scales are different from what is observed in our model. Both reported a linear decay over a distance of several millimeters in the monkey visual cortex. In our model we observe linear decay for tuned input as well, but correlations vanished after a few ten pixels which corresponds to about 400 µm in cat visual cortex (see Figure 6, with 1 px corresponding to 15 µm in our scaling).

In addition, Rosenbaum et al. (2017) also found sinusoidal decay of correlations after removing latent correlations using Gaussian process factor analysis. A full oscillation cycle was observed in about 5 mm in the monkey data as well as in their spiking neuron simulation (see also the last paragraph of Section 4.1.2). In comparison, in our model we measured a full oscillation for a blank stimulus within the first 50 pixels (c.f. Figure 4B). This result corresponds to less than a millimeter in cat visual cortex^3^.

Ch'ng and Reid (2010) observed a sinusoidal modulation of noise correlations with distance for spontaneous activity in the visual cortex of rats using two-photon imaging. In cat visual cortex Ch'ng and Reid (2010) only reported a seemingly exponential decay. However, they could measure noise correlations for a maximum pair distance of only 400 µm. Our model simulations imply that only beyond this distance one should expect an increase in correlations due to a sinusoidal modulation. Therefore, Ch'ng and Reid (2010) might have measured only the first quarter of a cycle of the spatial oscillation.

The emergence of spatially inhomogeneous patterns of spontaneous activity on the scale of several hundred micrometers has been long known (Arieli et al., 1995). For instance, Kenet et al. (2003) reported bump shaped spontaneous activity in the visual cortex of anesthetized cats using voltage sensitive dyes. More important, the spatial scales of the patterns, i.e., the size of the bumps, matched the distances of pinwheel centers of the preferred orientation map. They measured about one bump per pinwheel. This is also the case in our simulations. In our 2D network model we measured a spatial autocorrelation of about 2.2 cycles per 100 neurons, which agrees with the frequency of 4 pinwheels per 100 × 100 cells. This means we also observed about one bump per pinwheel.

#### 4.1.4. Limitations

The qualitative comparison of our model with biological data should, however, be taken with care because of our assumption of conditionally independent afferent Poisson input. This afferent input is not consistent with realistic LGN input. For instance, Lin et al. (2012) demonstrated that more realistic LGN models can change response characteristics such as tuning sharpening of post-synaptic V1 neurons. In this manuscript, however, we study the effect of recurrent connectivity in isolation and excluded other sources of correlated variability.

In our model all cells of a population exhibited equal parameter values (except those for coupling). In biology, however, one can observe a large variability among neuronal parameters such as membrane and adaptation time constants (Sanchez-Vives et al., 2000) or for the amplitudes of spike triggered post-synaptic potentials (Mason et al., 1991). A modeling study by Mejias and Longtin (2012) suggests that such heterogeneities can even enhance information processing in neural networks.

Furthermore, in our 2D networks with 100 × 100 and 200 × 200 excitatory neurons corresponding to 1.5 mm × 1.5 mm or 3 mm × 3 mm of cortical area, respectively, we obtain an average number of about 5,500 neurons per square millimeter (including inhibitory neurons). This is much less than the number of 15,000 neurons per square millimeter estimated for layer 4 (Beaulieu and Colonnier, 1983). Yet, the theory by Rosenbaum and Doiron (2014) states that increasing the network size by keeping the spatial extent constant even fosters inhomogeneous activity and stable Mexican hat networks may yield pattern formation of spiking activity due to a vanishing finite size effect. Accordingly, we could also demonstrate (in the Supplementary Material) that larger neuron numbers per extent yielded correlated variability for even more parameter settings.

We assumed periodic boundary conditions. Yet, we do not believe that our results would change considerably using other boundary conditions. We tested reflecting as well as absorbing boundary conditions in one-dimensional networks (see Supplementary Material) and observed the formation of similar patterns including the moving bump structures.

### 4.2. Neural coding

We demonstrated that noise correlations observed for Mexican hat topologies decrease the Fisher information in comparison to shuffled data where correlations have been removed. However, in comparison to other topologies like balanced or inverse Mexican hat, more information about the stimulus could be extracted even if correlations were present (cf. Figure 8) as long as a subset of neurons were considered for readout.

#### 4.2.1. Sharpening of orientation tuning

The sharpening of the orientation tuning due to the Mexican hat configuration may explain this relative gain in information in comparison to other topologies for neuron sub-sampling. Yet, as number of readout neurons increased, the deteriorating effect of correlation worsened and eliminated the advantage of tuning sharpening. Whether the improvement of coding of a sample readout population in terms of Fisher information carries over to situations where neurons are tuned to more than one feature, however, remains unclear. For stimulus feature spaces with more than two dimensions and uncorrelated neural firing, sharpening of feature tuning yields a reduction in Fisher information (Zhang and Sejnowski, 1999; Dayan and Abbott, 2005).

#### 4.2.2. Comparison to other modeling studies

Similar to our study Seriès et al. (2004) investigated the influence of noise correlations and sharpening of tuning curves on stimulus information. They used two different models. In the first model tuning was sharpened by strong recurrent Mexican hat connectivity much like in our model. In the second model the tuning of the afferent input was already sharp and no excitatory recurrent connections were present. The afferent inputs were chosen such that the output tuning curves of the two network models matched. In this setting, the Mexican hat network performed much worse than the model with afferent tuning. Besides a strong reduction in information due to noise correlations, Mexican hat networks showed already a much lower value of Fisher information for shuffled data where correlations were removed. However, Seriès et al. (2004) used a Mexican hat defined over orientation space with a very large inhibitory spread of σ_*I*_ = 60° compared to a very narrow excitatory width of σ_*E*_ = 7.5°. In our simulations we discovered that very wide inhibitory spreads can have devastating effects on the encoding quality. Information was enhanced only for a particular regime of Mexican hats (see Figure 10). Thus, the wide inhibitory spread used by Seriès et al. (2004) might explain why already shuffled information was low in their Mexican hat network.

Likewise, Hansen et al. (2012) developed a model with a Mexican hat defined over orientation space. However, their ratio between excitatory and inhibitory widths was smaller (σ_*E*_ = 15° and σ_*I*_ = 40°). Accordingly, in comparison to the work by Seriès et al. (2004), less Fisher information was lost in Mexican hat networks relative to topologies with wider excitatory spread.

Moreno-Bote et al. (2014) demonstrated analytically and numerically with networks of leaky integrate-and-fire neurons that a particular type of correlations is detrimental to stimulus encoding. They termed these *differential correlations*. The authors showed that the noise covariance matrix ***Q*** of neural responses to a stimulus *s* can be decomposed as:

(8) $$\begin{array}{l} \text{Q}\left(s\right)={\text{Q}}_{0}\left(s\right)+\varepsilon \text{f}\prime \left(s\right){\text{f}\prime }^{T}\left(s\right), \end{array}$$

where ***Q***_0_ represents noise that is not harmful to encoding, whereas correlations that are detrimental can take up the form ε ***f***′(*s*) ***f***′^*T*^(*s*). ε is a potentially small coefficient. ***f***′ denotes the derivative of the neural tuning curve vector with respect to stimulus *s*, and ***f***′^*T*^ its transpose. Hence, correlations are limiting encoding quality if they shift joint neural responses tangentially along the stimulus manifold in the neural response space. More simply, assuming a one-dimensional stimulus, like orientation, and a network response in form of a Gaussian curve or a bump profile defined over the stimulus space, the following holds: If noise moves the response curve back and forth across the stimulus space, this yields differential correlations and one cannot discriminate the noise from the actual stimulus. Indeed, we made similar observations in our network model with heterogeneous stimuli. The heterogeneous input locked bump activity to a particular location, but we still observed small jitter around the location of maximum afferent input (cf. Figure 7). Consequently, in our 2D networks featuring an orientation map, small movements of the bump responses defined over the two-dimensional neural space simultaneously imply jitter of network responses in the stimulus space because the preferred orientations of neurons smoothly change along the two spatial axes.

Similarly, the spatial profile of the differential correlations found by Moreno-Bote et al. (2014) are reminiscent of a sinusoidal modulation whose amplitude decays with distance in stimulus space akin to our results (cf. Figure 4A). The authors argued that differential correlations are small (ε ≪ 1) compared to other correlations (***Q***_0_), which makes them difficult to identify by simply measuring correlation coefficients. In order to detect differential correlations the authors suggested to use the decoder approach of Seriès et al. (2004) to estimate Fisher information. Accordingly, in case Fisher information is reduced in comparison to shuffled data, differential correlations are present. This is what we observed in our simulations (cf. Figure 8).

Kanitscheider et al. (2015) developed a generative model of differential correlations based on convergent feed-forward projections in a primary visual cortex model. In this model noise correlations emerged due to shared noise from LGN input to V1. The convergence of afferent inputs onto their V1 network model gave rise to shared input among their V1 model neurons. This produced correlations among the input currents which in turn led to correlations among the spiking output of the model neurons. In contrast, one can interpret our findings as a model of differential correlations originating from recurrent processing instead of afferent input. In our model recurrent connections enable noise and adaptation to cause a drift of the network activity representing an orientation stimulus, which in turn yields differential correlations among neuron pairs.

#### 4.2.3. Biological relevance of the results

A modulation of information due to noise correlations has been reported frequently in visual cortex experiments (Gu et al., 2011; Chelaru and Dragoi, 2014). Similar to our observations that noise correlations are less disadvantageous if neurons within a network were sub-sampled (cf. Figure 11), Montijn et al. (2014) measured a saturation of decoding performance with sample size using a variety of decoders in mouse visual cortex. Similar observations were made by Freiwald et al. (2002) reconstructing stimuli with a Bayesian decoder from data recorded in rat primary visual cortex. Comparable to our Mexican hat networks (cf. Figure 8), Graf et al. (2011) discovered that correlations among neurons in macaque primary visual cortex carry a significant amount of information. They reported that decoding accuracy could drop by more than five percent if a decoder ignored correlations.

Hansen et al. (2012) as well as Smith et al. (2013) found that the magnitude of correlations in monkey primary visual cortex is laminar dependent. For the input layer, often referred to as the granular (Hansen et al., 2012) or middle layer (Smith et al., 2013), the measured average *r*_*SC*_ was almost 0. Whereas in the deep or infra-granular (IG) as well as the superficial or supra-granular (SG) layers the experimenters measured significant correlations on the order of 0.1–0.2 for cells up to 300 µm apart. This can give rise to the interpretation that correlations are predominant in layers that are projecting to higher cortical areas. The output layers (IG and SG) use other stimulus encoding strategies that are less accurate in comparison to the input layer. In contrast, the middle layer, receiving input from the LGN, and projecting only to other layers in V1, provides a very accurate and unmodified representation of a stimulus.

## Author contributions

RM and KO designed the study. JL contributed to the final concept. RM implemented the network model and analyzed the simulation results. All authors wrote the manuscript.

## Funding

This work was funded by the Deutsche Forschungsgemeinschaft (GRK 1589/1, SFB 910).

### Conflict of interest statement

The authors declare that the research was conducted in the absence of any commercial or financial relationships that could be construed as a potential conflict of interest.

## Footnotes

^1^Model available on http://www.ni.tu-berlin.de/menue/software_and_data/visual_cortex_model, direct link to github: https://github.com/nigroup/visualcortex.

^2^To verify this method, periods of 9 s were tested as well and the results were similar.

^3^Note the scale difference in network extents and parameterization between our and their model is on the order of the difference in the spatial modulation of the correlations. For example, in our model the excitatory recurrent connection had a spread corresponding to σ_*E*_ = 150 µ*m* whereas Rosenbaum et al. (2017) used σ_*E*_ = 500 µ*m*.
