# Supplementary material for: The Influence of Mexican Hat Recurrent Connectivity on Noise Correlations and Stimulus Encoding
Source: Front Comput Neurosci. 2017 May 10;11:34. doi: 10.3389/fncom.2017.00034 (PMC5423970; doi:10.3389/fncom.2017.00034)
Supplement: Supplementary file 5 [file DataSheet1.pdf]

Supplementary Material: The Influence of Mexican Hat Recurrent Connectivity on Noise Correlations and Stimulus Encoding

Robert Meyer [1,2*], **Josef Ladenbauer** [1,2,3], **Klaus Obermayer** [1,2]

[1] *Department of Software Engineering and Theoretical Computer Science, Technische Universität Berlin, Berlin, Germany*
[2] *Bernstein Center for Computational Neuroscience, Berlin, Germany*
[3] *Group for Neural Theory, Laboratoire de Neurosciences Cognitives, École Normale Supérieure, Paris, France*

Correspondence*:
Robert Meyer
Technische Universität Berlin, Sekretariat MAR 5-6, Marchstrasse 23, 10587 Berlin, Germany, robert.meyer@ni.tu-berlin.de

Here we present the neural network model in depth including the values of all parameters. In addition, we provide some results for one-dimensional networks.

1 MODEL DETAILS

We used adaptive exponential integrate and fire neurons (AEIF) (**Brette and Gerstner**, 2005). Each neuron of population $X \in \{E, I\}$ indexed by i comprised two differential equations describing the development of the membrane potential $V_{X,i}$ and an adaptation current $w_{X,i}$ over time:

$$C\frac{dV_{X,i}(t)}{dt} = -g_{L,X}(V_{X,i} - E_L) + g_{L,X}\Delta_T \exp\left(\frac{V_{X,i} - V_T}{\Delta_T}\right) - w_{X,i} - I_{X,i}(t), \tag{1}$$

$$I_{X,i}(t) = I_{XA,i}(t) + I_{XE,i}(t) + I_{XI,i}(t), \tag{2}$$

$$\tau_w\frac{dw_{X,i}(t)}{dt} = a_X(V_i - E_L) - w_{X,i}, \tag{3}$$

with $-g_{L,X}(V_{X,i} - E_L)$ defining the leak term and $g_{L,X}\Delta_T \exp\left(\frac{V_{X,i}-V_T}{\Delta_T}\right)$ being an approximation to exponential rise of the sodium (Na^+) current of an action potential, assuming that the activation of Na^+-channels is instantaneous and inactivation can be neglected (**Fourcaud-Trocme et al.**, 2003). Moreover, C denotes the capacitance, $g_{L,X}$ is the leak conductance of population X, E_L the leak reversal potential, V_T the threshold parameter, Δ_T the slope factor, a_X the sub-threshold adaptation of population X, τ_w the adaptation time constant, and $I_{XY,i}$ denotes the afferent and recurrent currents. Moreover, each neuron

exhibited a threshold condition:

$$\text{if } V_{X,i} \geq V_{\text{cut}}: \quad V_{X,i} \leftarrow E_L \quad \text{(with clamping for } \tau_{\text{refr},X}), \qquad w_{X,i} \leftarrow w_{X,i} + b_X, \tag{4}$$

i.e. each neuron was reset to the leakage potential in case the membrane potential rose beyond the cut off point V_{cut}. In addition, this crossing was considered to be the particular point in time a neuron spiked. Then $w_{X,i}$ was increased by a constant term b_X, modeling spike-triggered adaptation. Moreover, the dynamic of $V_{X,i}$ was clamped for a period of $\tau_{\text{refr},X}$ to E_L after the crossing to simulate an absolute refractory period.

Note that we used different refractory periods $\tau_{\text{refr},X}$ for excitatory and inhibitory neurons. We assumed a shorter period for fast spiking inhibitory neurons of 2 ms in comparison to the excitatory cells with 3 ms. Similarly, we used different leak conductances $g_{L,X}$ among the two populations to account for the experimental observation that the conductance is about twice as large in inhibitory cells (**Karayannis et al.**, 2007).

Furthermore, the currents $I_{XY,i}$ with $Y \in \{A, E, I\}$ were modeled as conductance based:

$$I_{XY,i}(t) = g_{XY,i}(t)\,(V_{X,i}(t) - E_Y). \tag{5}$$

Afferent currents were mediated by fast exponentially decaying *AMPA*-like synapses. Recurrent input consisted of excitatory as well as an inhibitory part. The excitatory current depended on a mixture of fast *AMPA* and slow bi-exponential *NMDA*-like synapses, whereas the inhibitory current was based on fast *GABA*$_A$-like synapses:

$$\tau_{\text{AMPA}} \frac{dg_{XA,i}(t)}{dt} = -g_{XA,i}(t) + \bar{g}_{XA} \sum_{t_j^k} \delta(t_j^k - t), \tag{6}$$

$$g_{XE,i}(t) = g_{XE_{\text{AMPA}},i}(t) + g_{XE_{\text{NMDA}},i}(t), \tag{7}$$

$$\tau_{\text{AMPA}} \frac{dg_{XE_{\text{AMPA}},i}(t)}{dt} = -\,g_{XE_{\text{AMPA}},i}(t) + \alpha\,\bar{g}_{XE} \sum_{t_j^k} \delta(t_j^k - t + \Delta_{ij}), \tag{8}$$

$$\tau_{\text{decay}} \frac{dg_{XE_{\text{NMDA}},i}(t)}{dt} = -\,g_{XE_{\text{NMDA}},i}(t) + \beta\,h_{XE_{\text{NMDA}},i}(t), \tag{9}$$

$$\tau_{\text{rise}} \frac{dh_{XE_{\text{NMDA}},i}(t)}{dt} = -\,h_{XE_{\text{NMDA}},i}(t) + (1-\alpha)\bar{g}_{XE} \sum_{t_j^k} \delta(t_j^k - t + \Delta_{ij}), \tag{10}$$

$$\beta = \left(\frac{\tau_{\text{rise}}}{\tau_{\text{decay}}}\right)^{\tau_{\text{decay}}/(\tau_{\text{rise}}-\tau_{\text{decay}})}, \tag{11}$$

$$\tau_{\text{GABA}_\text{A}} \frac{dg_{XI,i}(t)}{dt} = -\,g_{XI,i}(t) + \bar{g}_{XI} \sum_{t_j^k} \delta(t_j^k - t + \Delta_{ij}), \tag{12}$$

where $\alpha \in [0, 1]$ determines the ratio between *AMPA* and *NMDA* receptors and t_j^k is the time point of the kth spike of pre-synaptic neuron j. In addition, $\bar{g}_{XY}$ denotes the maximum conductance, i.e. the synaptic coupling strength, $\tau_{\dots}$ are time constants, E_Y is the synaptic reversal potential, and Δ_{ij} is the synaptic delay between neuron j and i as given below.

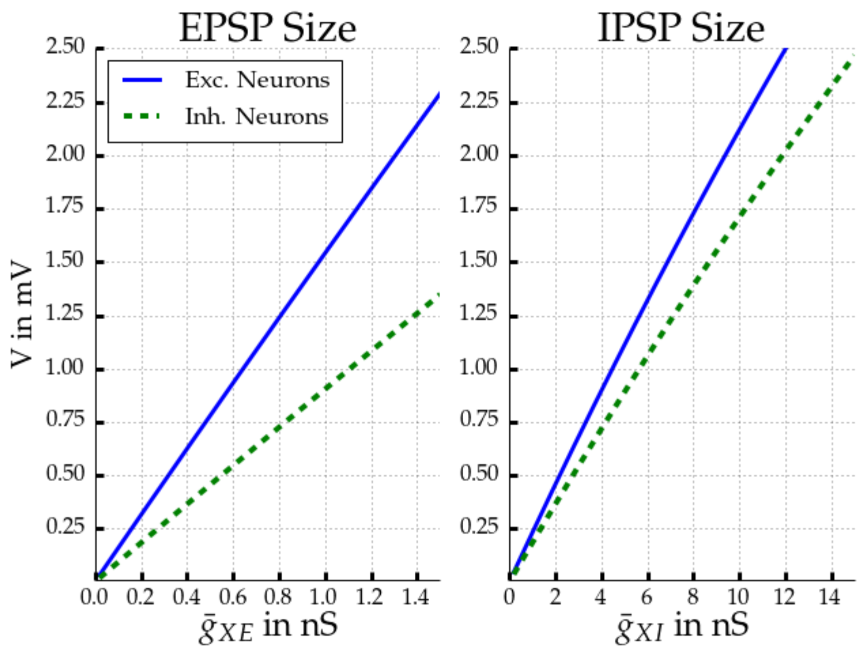

Figure 1: Maximum absolute deflection of membrane voltage at resting potential (-65 mV) for single pre-synaptic spikes as function of connection strength. Excitatory post-synaptic potential (EPSP) size on the left and inhibitory post-synaptic potential (IPSP) on the right; in blue for excitatory neurons and inhibitory neurons in dotted green.

1.1 SYNAPTIC DELAYS

Synaptic delays Δ_{ij} were distance dependent and computed as

$$\Delta_{ij} = \underline{\Delta}_{ij} + \frac{d_{ij}}{v}, \tag{13}$$

with a basic time delay $\underline{\Delta}_{ij}$ that was sampled uniformly from the interval $[0.2, 1.2]$ ms. Moreover, v denotes the signal velocity and d_{ij} is the distance between cells i and j. We assumed a signal velocity of $v = 200\,\mathrm{kpx/s}$ for the one-dimensional networks (see below) and $v = 13.3\,\mathrm{kpx/s}$ in the two-dimensional setting which corresponds to 0.2 m/s in our cat cortex scaling.

1.2 NUMERICAL SIMULATION

We integrated all equations using a simple Euler scheme with a particular step size dt. The runtime was optimized by pre-compiling Python code into faster C code using the Numba package (**The Numba Development Team**, 2015).

To allow fast computation of the exponential upstroke as in $g_{L,X}\Delta_T \exp\left(\frac{V_i(t)-V_T}{\Delta_T}\right)$, we precomputed the function. We used 10^6 sampling points and linearly sampled membrane voltages in a range between 92.5 mV and V_{cut}. We did not interpolate in between points. For membrane voltages falling in between two sampling points, we used the result of the closest point. For values smaller than the lower bound, we simply took a voltage of 92.5 mV.

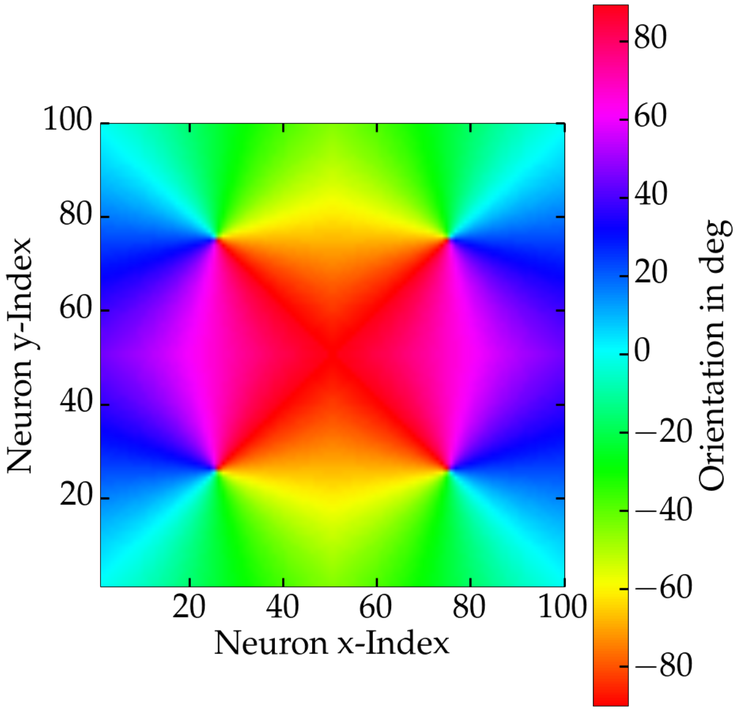

Figure 2: Pinwheel Map

Randomly sampled membrane potentials $V_{X,i}$ from a uniform distribution over the interval $[E_L, V_T]$ were used as initial conditions. All other dynamic parameters were set to 0. Moreover, before any measures or statistics were computed a simulation was run for at least 1 second to allow the network to settle into a stationary state.

The source code is publicly available and hosted on github (`https://github.com/nigroup/visualcortex`).

2 PARAMETER SETTINGS

In here we provide all parameter settings, see table 1. The relation between potential sizes and recurrent connections strength is shown in figure 1. The pinwheel map of the heterogeneous input for the 2D networks is also depicted in figure 2, showing the preferred orientations s_{PO_i} of 100×100 neurons.

Parameter	Description	Default Value	varied
Simulation			
dt	Euler step size	0.1 ms	
Network Topology			
N_E	Number of excitatory neurons	10,000	yes
N_I	Number of inhibitory neurons	2500	yes
K_E	Number of excitatory connections	200 (1D) or 400 (2D)	in supplementary
K_I	Number of inhibitory connections	100 (1D) or 200 (2D)	in supplementary
K_A	Number of afferent connections	100	in supplementary
D	Map dimensionality	1 or 2	
σ_E	Excitatory connection spread	125 (1D) or 10 px (2D)	yes
σ_I	Inhibitory connection spread	250 (1D) or 15 px (2D)	yes
$\underline{\Delta}_{ij}$	Basic synaptic delay	[0.2, 1.2] ms	
v	Signal velocity	200 (1D) or 13.3 kpx/s (2D)	
Neuron Properties			
C	Capacitance	200 pF	
$g_{L,E}$	Excitatory leak conductance	10 nS	
$g_{L,I}$	Inhibitory leak conductance	20 nS	
E_L	Leak reversal potential	–65 mV	
V_T	Threshold parameter	–50 mV	
Δ_T	Slope factor	2 mV	
V_{cut}	Spike cut-off	–30 mV	
$\tau_{\text{refr},E}$	Excitatory refractory period	3 ms	
$\tau_{\text{refr},I}$	Inhibitory refractory period	2 ms	
τ_w	Adaptation time constant	250 ms	
a_E	Exc. sub-threshold adaptation	2 nS	
a_I	Inh. sub-threshold adaptation	0.2 nS	
b_E	Excitatory SFA strength	50 pA	
b_I	Inhibitory SFA strength	5 pA	
Synaptic Coupling			
$\bar{g}_{EE}$	Exc. to exc. coupling strength	0.4 nS	yes
$\bar{g}_{IE}$	Exc. to inh. coupling strength	0.6 nS	yes
$\bar{g}_{EI}$	Inh. to exc. coupling strength	5.0 nS	
$\bar{g}_{II}$	Inh. to inh. coupling strength	5.0 nS	
$\bar{g}_{EA}$	Aff. to exc. coupling strength	1.5 nS	
$\bar{g}_{IA}$	Aff. to inh. coupling strength	0.5 nS	
E_E	Excitatory reversal potential	0 mV	
E_I	Inhibitory reversal potential	–80 mV	
E_A	Afferent reversal potential	0 mV	
α	Fraction of AMPA receptors	0.7	
τ_{AMPA}	AMPA decay time constant	3 ms	
τ_{rise}	NMDA rise time constant	5 ms	
τ_{decay}	NMDA decay time constant	80 ms	

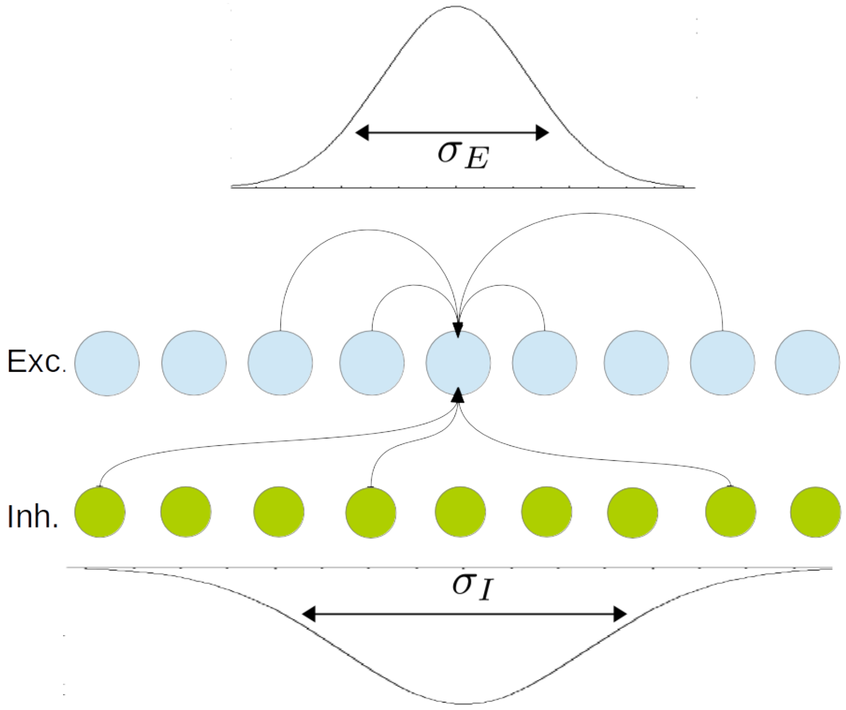

Figure 3: One-dimensional network realization with an excitatory as well as inhibitory population. Recurrent connectivity are sampled from two Gaussian with widths σ_E and σ_I with periodic boundary conditions.

**Parameter**	**Description**	**Default Value**	**varied**
$\tau_{\mathrm{GABA_A}}$	$\mathrm{GABA_A}$ decay time constant	5 ms	
Homogeneous Input			
$\nu_{\mathrm{Aff,i}}$	Afferent firing rate	15 Hz (1D) or 10 Hz (2D)	yes
Heterogeneous Input 2D			
$\nu_{\mathrm{Aff,\,max}}$	Maximum afferent firing rate	30 Hz	yes
$\nu_{\mathrm{Aff,\,base}}$	Baseline afferent firing rate	3 Hz	
σ_{Aff}	Input tuning width	27.5°	
s	Orientation stimulus	1°	yes
s_{PO_i}	Preferred orientation	See figure 2	

Table 1: Model Parameters. All parameters that were systematically varied at some point are marked as such in the last column.

3 NOISE CORRELATIONS IN ONE-DIMENSIONAL NETWORKS

We turned to simulations of ring networks similar to the model by **Hansen et al.** (2012) to test our hypothesis in a simpler topology as well. Such a one-dimensional model is depicted in figure 3.

Our networks consisted of $N_E = 10{,}000$ excitatory and $N_I = 2500$ inhibitory neurons placed on a ring with a length scale of 10 kpx. Due to periodic boundary conditions the maximum distance between two cells is therefore 5 kpx. Each neuron received $K_E = 200$ excitatory and $K_I = 100$ inhibitory recurrent inputs. In this one-dimensional setting we only considered homogeneous input of 15 Hz with $K_A = 100$ afferent connections per cell.

In figure 4A the spike count correlation coefficient as a function of distance between cell pairs is shown for a Mexican hat network close to self-sustained activity ($\sigma_E = 125\,\text{px} < \sigma_I = 250\,\text{px}$, $\bar{g}_{EE} = 0.35\,\text{nS}$, $\bar{g}_{IE} = 0.6\,\text{nS}$). Each blue dot corresponds to the r_{SC} value for an individual cell pair calculated over 50 stimulus presentations of 1 second length. For comparison the noise correlations of an inverse Mexican hat ($\sigma_E = 250\,\text{px} > \sigma_I = 125\,\text{px}$) are shown in the small inset. No distance dependency of correlations was observed in this case.

The distance dependency of spike count correlations in one-dimensional Mexican hat networks were fitted using an exponentially decaying sine wave with

$$\langle r_{SC}\rangle(d) = C_{r_{SC}} \exp(-|d|/\lambda_{r_{SC}}) \cos(\omega_{r_{SC}}\, d), \tag{14}$$

where $\langle r_{SC}\rangle(d)$ is expectation across neuron pairs with the same distance, $C_{r_{SC}}$ is the correlation fit for $d = 0$, $\lambda_{r_{SC}}$ is the mean decay lifetime, and $\omega_{r_{SC}}$ denotes the spatial frequency. The fits were calculated using curve fitting from SciPy's optimization library (**Jones et al.**, 2001). This optimization was repeated 10 times with random initial values and the parameters of the best fit were chosen.

The average value for bins of 20 px is plotted in green in figure 4A. For the Mexican hat network this average correlation coefficient can be neatly fitted with a damped sinusoidal wave (see equation 14); shown on top of the green curve in red. For close by cells correlation is relatively large and we fitted an initial value $C_{r_{SC}} \approx 0.64$. Furthermore, the exponential decay was quite slow with $\lambda_{r_{SC}} \approx 3.4\,\text{kpx}$. Lastly, the spatial frequency was fast with $\omega_{r_{SC}} \approx 1.4$ per thousand pixels.

Moreover, we investigated whether the spatial scales of the noise correlations correspond to the spatial scales of the moving bumps. The average spatial Fourier spectrum alongside the average spatial autocorrelation is shown in figures 4C and D, respectively. Indeed, the highest power was measured for 1.4 cycles per thousand pixels, which nicely fits the spatial scale of the noise correlations. Moreover, the spatial power spectrum does not show a spiky peak at only 1.4 cycles, but a slight broadening around this value. This matches the observations one makes looking directly at the network activity. Usually we counted 14 bumps within the entire network of 10,000 neurons, but occasionally due to splits and merges of bumps one more or less could be found.

Furthermore, we observed an increase of correlated variability with increase in the size of the integration window. As seen in figure 5A, longer integration time windows yielded larger amplitudes of the damped sine wave. Moreover, increasing the integration window beyond 1 second yielded a saturation of average correlation of close by cells as depicted in figure 5B. We recall that the noise correlation coefficient is defined as the ratio between the covariance and the product of individual neurons' standard deviations of spike counts. In this line of thought, we took a look at the relation between the mean covariance of nearby neurons and the geometric mean spike count variance of individual neurons. The results are depicted in figure 5D. We observed that for smaller window sizes the covariance was much smaller in comparison to the variance. Hence, much of the cells' covariation was hidden by the spiking threshold.

Moreover, as shown in the temporal power spectrum (figure 5C), the movement of the bumps was rather slow. This is indicated by the pronounced power in the low frequency band. The peak of the temporal power spectrum was found for 0.2 Hz.

3.1 DEPENDENCY OF NOISE CORRELATIONS ON NETWORK PARAMETERS

We explored the dependency of the fitted parameters from equation 14 as a function of different connection strengths $\bar{g}_{EE}$ as well as different Mexican hat sizes in terms of inhibitory connection spread σ_I. The fitted parameters as well as some other basic statistics such as the average firing rate, the coefficient of variation (CV), and the average noise correlation coefficient (r_{SC}) for cells at most 100 px apart are shown in figure 6.

As one can see in figure 6A, the average firing rate increased slowly with increasing excitatory to excitatory strength until self-sustained activity is reached as depicted by the orange border. Beyond the border the average firing rate quickly diverged.

Moreover, figure 6B shows an increase of the coefficient of variation (CV) averaged across all excitatory cells with an increase of $\bar{g}_{EE}$. As figure 6B illustrates with increasing strength the average CV exhibited a rather sudden jump beyond 1 after which a seemingly exponential growth happened. Of note, the value of 1 is a standard reference point because it is also the CV for any homogeneous Poisson process. Hence, the spiking activity of the cells was more random and irregular than Poisson spiking. CVs larger than 1 are usually indicative of bursting activity (**Christodoulou and Clarkson**, 1995). Bursting neurons show periods of no activity followed by periods with strong repetitive firing. We could identify that such bursting behavior was indeed present for each individual cell by looking at the spike and voltage traces (data not shown). Yet, if one takes the joint neural activity into account, one can understand that the bursts are due to the moving activity bumps. Thus, cells may be participating in a bump, i.e. bursting, or are silent if they are part of an inactive region.

Figure 6C shows the noise correlation coefficient r_{SC} over 50 stimulus presentations using a time window of 1 second and averaged over cell pairs being at most 100 px apart. Accordingly, the coefficient is averaged among close by cells to get an estimate of the magnitude or initial amplitude of the distance dependent noise correlations. For lower connection strength $\bar{g}_{EE}$, the average noise correlation was essentially zero. For values $\bar{g}_{EE} \geq 0.2\,\text{nS}$ there was a rapid increase followed by a plateau around 0.4 close to the bifurcation to self-sustained activity and a further increase beyond this point. When inspecting the raster plots for small values of $\bar{g}_{EE} < 0.2\,\text{nS}$, we observed no bump activity. **Rosenbaum and Doiron** (2014) showed that the homogeneous fixpoint with asynchronous activity can still be stable even in case of Mexican hat coupling due to a finite network size. Indeed, this was the case here. We tested different network sizes, i.e. we increased the neuron density, and scaled the number of afferent and recurrent connections and the connection spread proportionally while keeping the connection strength fixed. Using low values of $\bar{g}_{EE} = 0.15\,\text{nS}$ and $0.2\,\text{nS}$, we observed an increase in noise correlations between neurons that are close by for larger network sizes, as depicted in figure 7.

The middle row (D-F) of figure 6 shows the fitted parameters for equation 14 for different connection strengths. The parameters were only fitted for $\bar{g}_{EE} \geq 0.2\,\text{nS}$.

With increase in connection strength, the frequency of the noise correlations $\omega_{r_{SC}}$ decreased slightly in figure 6D. Likewise, by looking at raster plots of the corresponding runs, we could observe a decrease in bump frequency, too (data not shown).

Furthermore, the relation between $\bar{g}_{EE}$ and $\lambda_{r_{SC}}$ was non-monotonic. First, in figure 6E we identified an increase in $\lambda_{r_{SC}}$ which means a slower decay of average correlations, followed by a decrease towards instability. Overall the exponential decay of correlations with distance was rather slow, similar to what was observed in figure 4A. Hence, there were even considerable positive and negative correlations between neurons with wide distances of 4000 or 5000 pixels apart.

As expected, the shape of the curve for the fitted initial correlations $C_{r_{SC}}$ in figure 6F strongly resembles the one above (figure 6C) showing the empirical average noise correlation coefficient r_{SC}. Since the empirical one is based on cell pairs at most 100 px apart, its magnitude is a bit lower than compared to $C_{r_{SC}}$. As a reminder, $C_{r_{SC}}$ fits the hypothetical average noise correlation value for cell pairs with no distance in between them.

The bottom row (G-I) of figure 6 displays the parameter fits as a function of inhibitory spread σ_I for a fixed connection strength of $\bar{g}_{EE} = 0.4\,\mathrm{nS}$. Similar to increasing connection strength, widening the inhibitory connection spread yielded a decrease in the spatial frequency of noise correlations (G). Surprisingly, there was no effect of inhibitory connection spread on the decay $\lambda_{r_{SC}}$ (H) and only a minor one on the amplitude parameter $C_{r_{SC}}$ (I). We observed a weak decrease in $C_{r_{SC}}$ for wider spreads.

3.2 BOUNDARY CONDITIONS

We further investigated the question: Do Mexican hat networks yield spatial activity patterns for other boundary conditions as well?

We ran simulations with absorbing as well as reflecting boundary conditions in one-dimensional networks. In the former case, neurons close to one of the boundaries received less inputs from their neighbors because outgoing connections beyond the boundary were simply cut off. In the latter case connectivity at the boundaries was denser than in the center of the network. Connections that were supposed to reach over the boundary were mirrored and projected back into the network.

Figure 8 shows network activity for these two different boundary conditions. As for circular boundary conditions, we observed the emergence of spatial patterns in Mexican hat networks close to self-sustained activity. In turn, these spatial patterns yielded noise correlations (data not shown). Likewise, no patterns and correlations were observed for balanced and inverse spreads (data not shown).

3.3 NOISE CORRELATIONS AND ADAPTATION

Clearly, adaptation plays an important role in the emergence of noise correlations. Adaptation yields movement of the bump solutions. Accordingly, removing adaptation from the spiking neurons reduced the magnitude of noise correlations for nearby neurons considerably (figure 9).

As shown by **Hansel and Sompolinsky** (1998) stronger adaptation in a sinusoidal rate model increases the bump velocity. We observed a similar behavior for the Gaussian Mexican hat coupling in the spiking neural networks. For the weak default SFA with $b_E = 50\,\mathrm{pA}$, we identified slow bump movements that eventually change directions. Whereas stronger adaptations like 100 or 200 pA yielded coordinated unidirectional bump movement as depicted in figure 10.

REFERENCES

Brette, R. and Gerstner, W. (2005), Adaptive exponential integrate-and-fire model as an effective description of neuronal activity., *J. Neurophysiol.*, 94, 5, 3637–3642, doi:10.1152/jn.00686.2005

Christodoulou, C. and Clarkson, T. (1995), A review on the stochastic firing behaviour of real neurons and how it can be modelled, in J. Mira and F. Sandoval, eds., From Nat. to Artif. Neural Comput. SE - 31, volume 930 of *Lecture Notes in Computer Science* (Springer Berlin Heidelberg), 223–230, doi:10.1007/3-540-59497-3_179

Fourcaud-Trocme, N., Hansel, D., Vreeswijk, C. V., and Brunel, N. (2003), How spike generation mechanisms determine the neuronal response to fluctuating inputs, *J. Neurosci.*, 23, 37, 11628–11640

Hansel, D. and Sompolinsky, H. (1998), Modeling feature selectivity in local cortical circuits, in Methods Neuronal Model. From Ions to Networks (MIT Press), 499–567

Hansen, B. J., Chelaru, M. I., and Dragoi, V. (2012), Correlated variability in laminar cortical circuits, *Neuron*, 76, 3, 590–602, doi:10.1016/j.neuron.2012.08.029

Jones, E., Oliphant, T., Peterson, P., and Others (2001), SciPy: Open Source Scientific Tools for Python

Karayannis, T., Huerta-Ocampo, I., and Capogna, M. (2007), GABAergic and pyramidal neurons of deep cortical layers directly receive and differently integrate callosal input, *Cereb. Cortex*, 17, 5, 1213–1226, doi:10.1093/cercor/bhl035

Rosenbaum, R. and Doiron, B. (2014), Balanced Networks of Spiking Neurons with Spatially Dependent Recurrent Connections, *Phys. Rev. X*, 4, 2, doi:10.1103/PhysRevX.4.021039
The Numba Development Team (2015), Numba Version 0.22.1

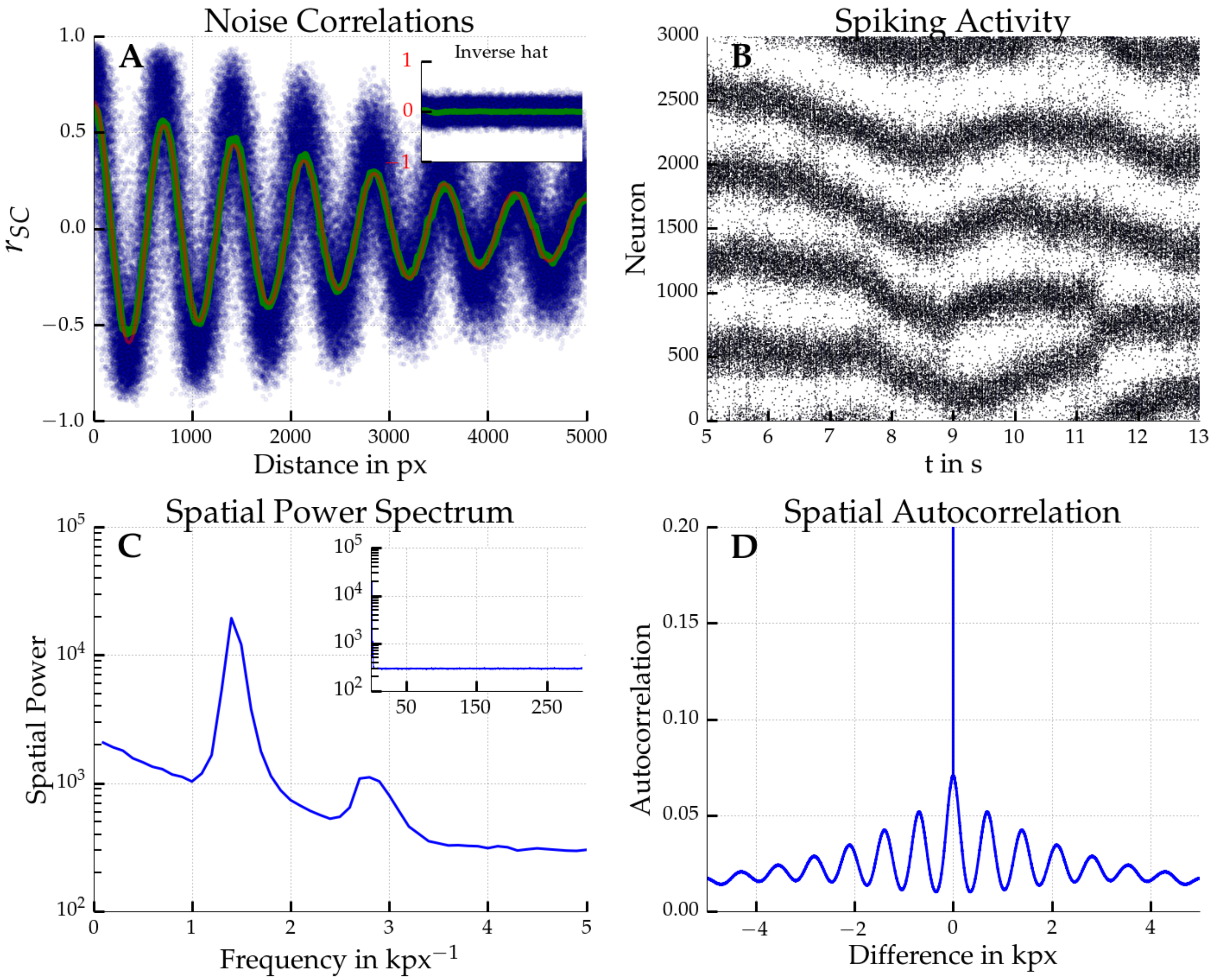

Figure 4: Top left (A): Noise correlations as a function of distance in a Mexican hat network ($\sigma_E = 125\,\mathrm{px} < \sigma_I = 250\,\mathrm{px}$, $\bar{g}_{EE} = 0.35\,\mathrm{nS}$, $\bar{g}_{IE} = 0.6\,\mathrm{nS}$). Blue dots represent r_{SC} of individual neuron pairs computed over a time window of 1 second and 50 stimulus presentations. One can observe a damped oscillation which can be nicely fit by equation 14. The fitted function is shown in red and tightly overlays the empirical average computed from the data in green. The inset shows the correlations for an inverse Mexican hat ($\sigma_E = 250\,\mathrm{px} > \sigma_I = 125\,\mathrm{px}$) with the same recurrent strengths. Top right (B): Spike raster plot of 5000 excitatory neurons. Bottom left (C): Spatial power spectrum computed from activity snapshots of 10 ms averaged over 5000 samples. The frequency with the highest power (1.4 cycles per kpx) nicely fits the oscillations of the noise correlations with distance (A). The power spectrum for higher spatial frequencies is shown in the inset and is basically flat. Bottom right (D): Average autocorrelation of the activity snapshots.

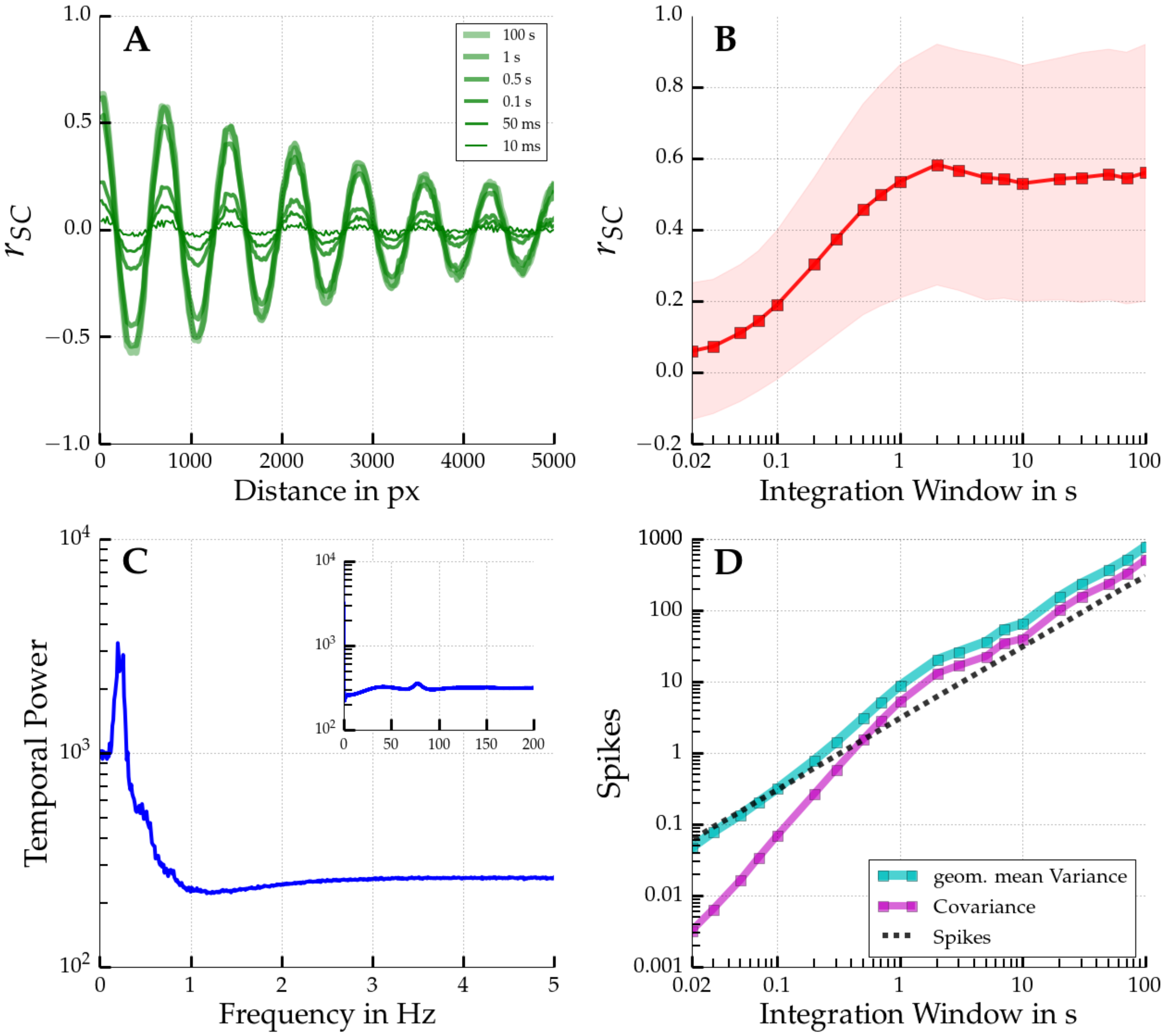

Figure 5: Temporal Scales of Noise correlations. Network parameters as in figure 4. Top left (A): Average noise correlation as a function of cell distance computed over 50 repeated stimulus presentations. Different integration time window sizes are shown with increasing thickness of the lines. The smallest window we use is 20 ms because for smaller windows only a tiny minority of cells exhibits at least a single spike within 50 stimulus presentations, which strongly biases our calculation of the spike count correlation coefficient. Increasing window size increases the amplitude of the damped wave. Top right (B): Average noise correlation for cells at most 100 px apart as a function of integration window size. This corresponds to less than the first fourth of a cycle of the damped sine waves one the top left. The envelope shows the standard deviation among all cell pairs. Increasing the window size beyond 1 second yields a saturation effect. Bottom left (C): Average temporal power spectrum. Movement of bumps happens on slow time scales as the peak and pronounced power on very low frequencies suggest. Additionally, there is another shallow peak in the gamma frequency band, as shown in the inset. Bottom right (D): Contribution of spike count variance and covariance to the average r_{SC} shown above. For smaller window sizes the covariance is much smaller than the neuron pairs' gemoetric mean variance of spike counts. For increasing window size the difference is reduced and both lines become parallel. The expected number of spikes per neuron is shown as a black dotted line.

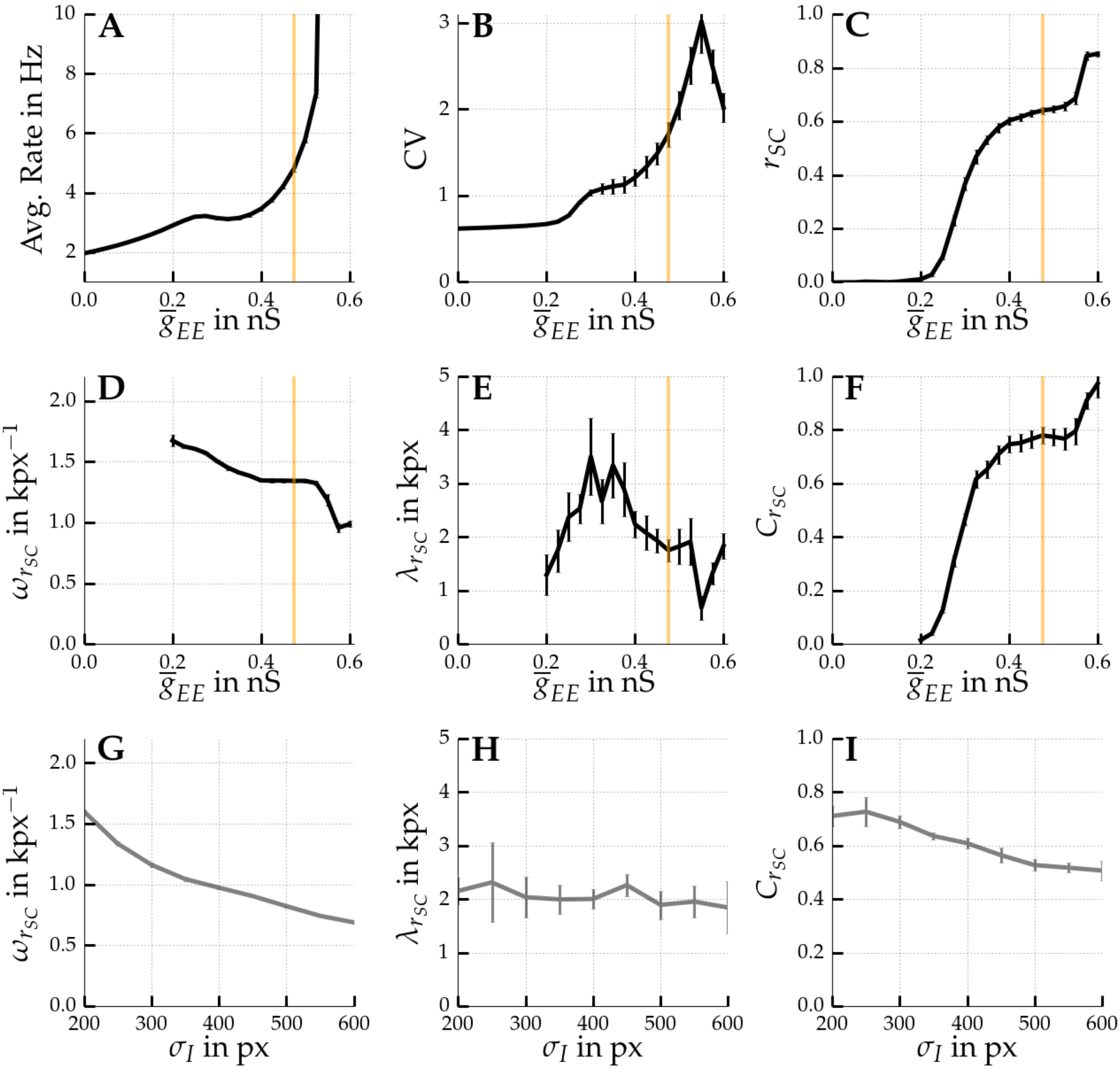

Figure 6: Top two rows (A-D) show parameter explorations of different excitatory to excitatory connection strengths. $\bar{g}_{EE}$ is explored using a Mexican hat topology with $\sigma_E = 125\,\text{px} > \sigma_I = 250\,\text{px}$, and $\bar{g}_{IE} = 0.6\,\text{nS}$. The orange line marks the bifurcation to self-sustained activity. All values are computed from 50 stimulus presentations and averaged across 10 network realizations. Values are computed from excitatory neurons only. Error bars mark the standard deviation across network realizations. The top row shows from left to right: (A) the average firing rate as a function of connection strength, followed by (B) the average coefficient of variation (CV), and (C) the average noise correlation coefficient (r_{SC}) for cells with at most 100 px distance between them. The r_{SC} is computed over an integration window of 1 second and based on 50 stimulus presentations. Middle row shows the curve parameters from fitting distance dependend noise correlation with equation 14. From left to right: (D) Average revolutions per thousand pixels ($\omega_{r_{SC}}$), (E) decay constant ($\lambda_{r_{SC}}$), and (F) maximum average noise correlation ($C_{r_{SC}}$). Values were computed only for $\bar{g}_{EE} \geq 0.2\,\text{nS}$ because for smaller values no distance dependent noise correlations were observed. Bottom row (G-I) same as in the middle but for exploring inhibitory connection spread σ_I using $\bar{g}_{EE} = 0.4\,\text{nS}$ and $\sigma_E = 125\,\text{px}$.

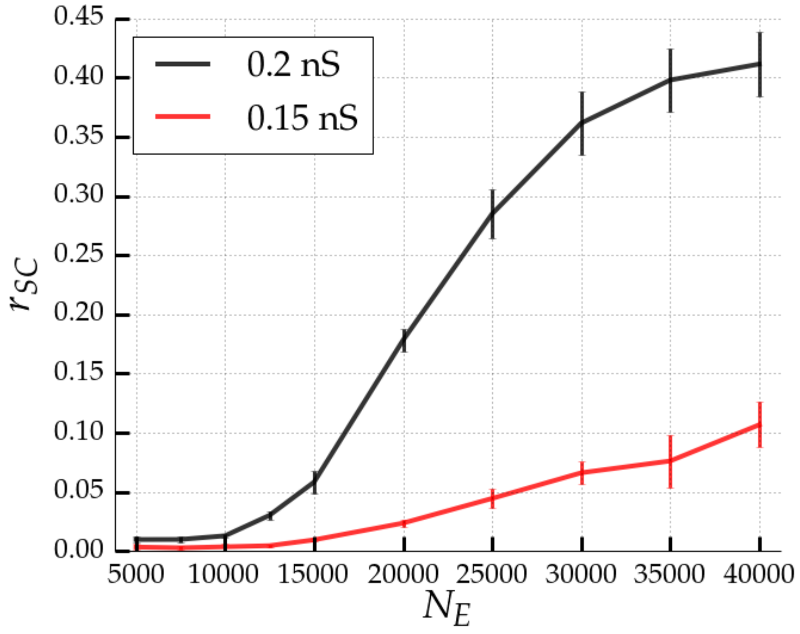

Figure 7: Average noise correlation as a function of network size. Correlations are computed and averaged from 50 stimulus presentations between neurons that are at most $k100\,\mathrm{px}$ apart with k the size factor, $k = N_E/10{,}000$. The connection strength is kept fixed $\bar{g}_{EE} = 0.15\,\mathrm{nS}$ or $0.2\,\mathrm{nS}$ and $\bar{g}_{IE} = 0.6\,\mathrm{nS}$. Spreads are $\sigma_E = 125\,\mathrm{px}$ and $\sigma_I = 250\,\mathrm{px}$. The number of inhibitory neurons (N_I) as well as the number of connections (K_A, K_E, and K_I) and the spread of connections (σ_E and σ_I) scale linearly with the number of excitatory neurons (N_E).

(a) Reflecting boundary

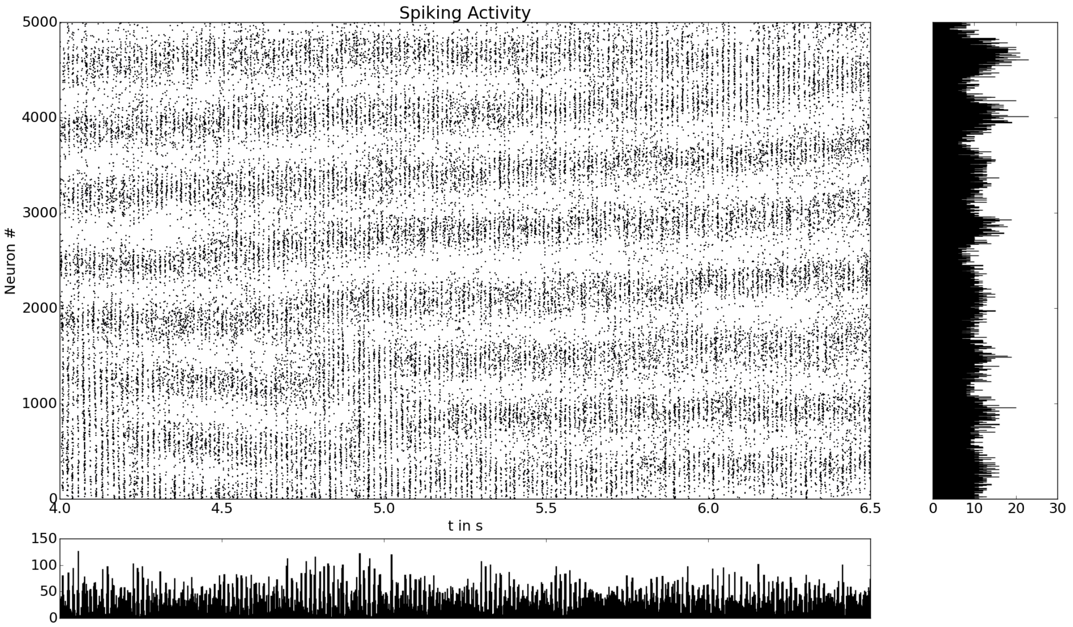

(b) Absorbing boundary

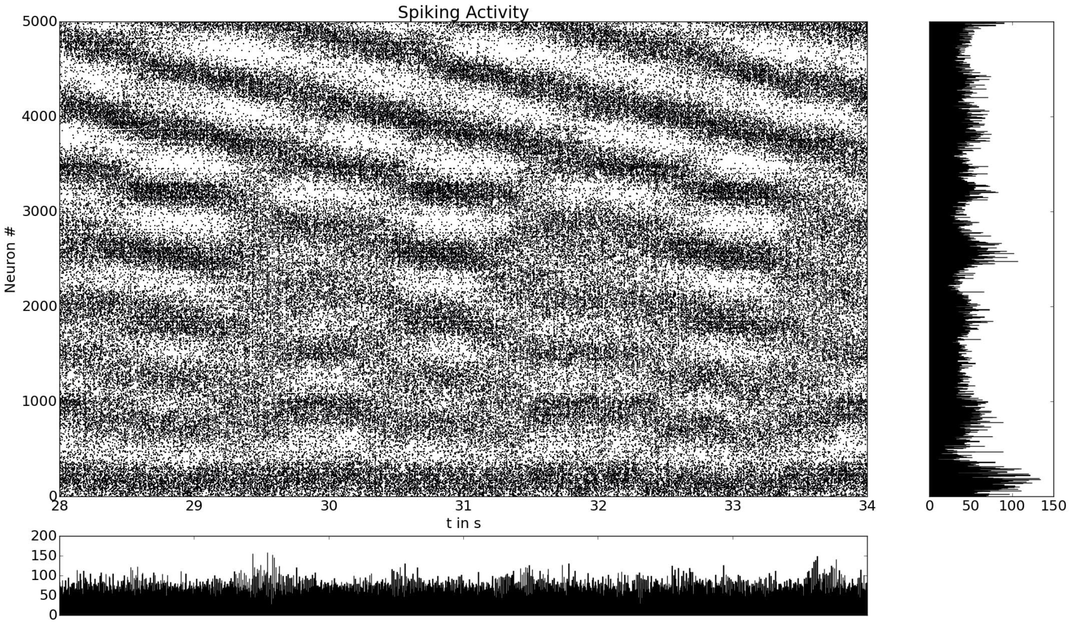

Figure 8: Spiking activity of a Mexican hat network ($\sigma_E = 125\,\mathrm{px} < \sigma_E = 250\,\mathrm{px}$, $\bar{g}_{EE} = 0.3\,\mathrm{nS}$, $\bar{g}_{IE} = 0.6\,\mathrm{nS}$) for reflecting (top) as well as absorbing (bottom) boundary conditions. A small black dot represents a spike of a particular neuron at a particular point in time. Neurons are ordered according to their position on the ring. Small plots show the activity histograms over time (horizontal) as well as over space (vertical). As for circular boundary conditions spatial patterns are observed.

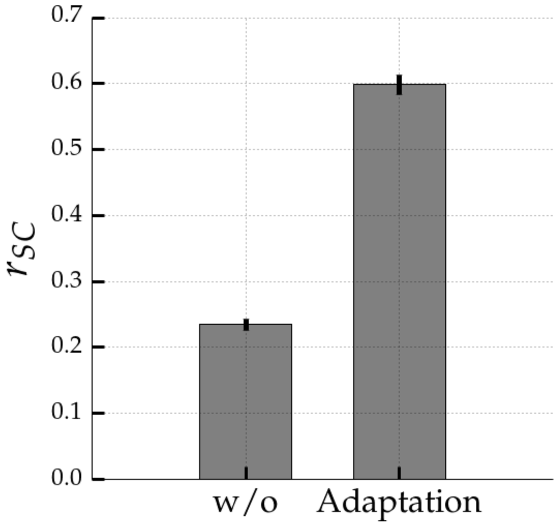

Figure 9: Average noise correlation within a Mexican hat network close to self-sustained activity ($\sigma_E = 125\,\mathrm{px} < \sigma_E = 250\,\mathrm{px}$, $\bar{g}_{EE} = 0.4\,\mathrm{nS}$, $\bar{g}_{IE} = 0.6\,\mathrm{nS}$). Values are averaged across pairs at most $100\,\mathrm{px}$ apart over 50 stimulus presentations of 1 second each with homogeneous input. Error bars mark standard deviations across 10 network realizations. On the left without adaptation $a_X = b_X = 0$ and on the right with adaptation $a_E = 10a_I = 2\,\mathrm{nS}$ and $b_E = 10b_I = 50\,\mathrm{pA}$. Removing adaptation significantly reduces correlations (Wilcoxon rank-sum test, $p < 0.001$, 10 network samples).

(a) $b_E = 50\,\text{pA}$

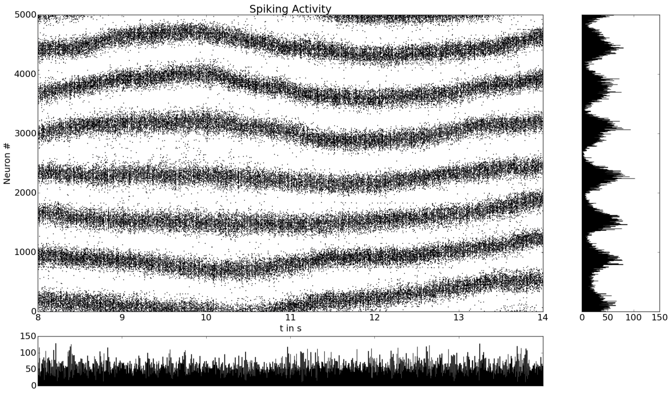

(b) $b_E = 100\,\text{pA}$

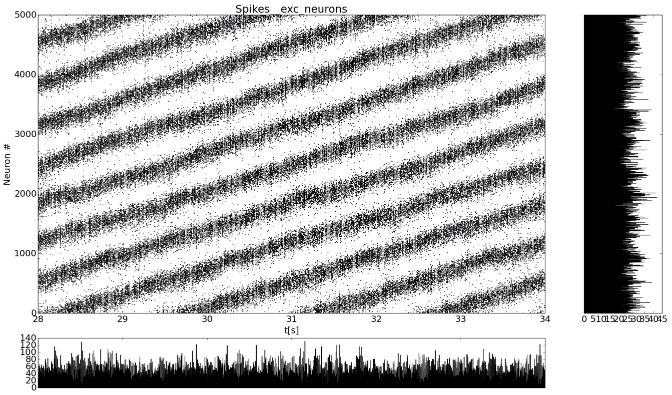

(c) $b_E = 200\,\text{pA}$

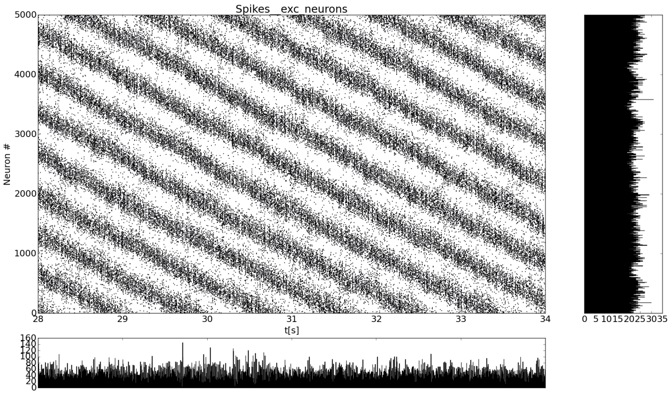

Figure 10: Spiking activity of a Mexican hat network ($\sigma_E = 125\,\text{px} < \sigma_E = 250\,\text{px}$, $\bar{g}_{EE} = 0.4\,\text{nS}$, $\bar{g}_{IE} = 0.6\,\text{nS}$) for three different SFA strengths b_E. A small black dot represents a spike of a particular neuron at a particular point in time. Neurons are ordered according to their position on the ring. Small plots show the activity histograms over time (horizontal) as well as over space (vertical).
